# Synthetic standard aided quantification and structural characterization of amyloid-beta glycopeptides enriched from cerebrospinal fluid of Alzheimer’s disease patients

**DOI:** 10.1038/s41598-019-41897-5

**Published:** 2019-04-02

**Authors:** Jonas Nilsson, Gunnar Brinkmalm, Sherif Ramadan, Lisa Gilborne, Fredrik Noborn, Kaj Blennow, Anders Wallin, Johan Svensson, Mohamed A. Abo-Riya, Xuefei Huang, Göran Larson

**Affiliations:** 1 Laboratory of Clinical Chemistry, Sahlgrenska University Hospital, Gothenburg, Sweden; 2 Department of Clinical Chemistry and Transfusion Medicine, Institute of Biomedicine, Sahlgrenska Academy, University of Gothenburg, Gothenburg, Sweden; 3 Department of Psychiatry and Neurochemistry, Institute of Neuroscience and Physiology, Sahlgrenska Academy, University of Gothenburg, Gothenburg, Sweden; 4 Departments of Chemistry and Biomedical Engineering, Institute for Quantitative Health, Science and Engineering, Michigan State University, East Lansing, MI USA; 5 Chemistry Department, Faculty of Science, Benha University, Benha, Qaliobiya 13518 Egypt; 6 Clinical Neurochemistry Laboratory, Sahlgrenska University Hospital, Mölndal, Sweden; 7 Department of Internal Medicine, Institute of Medicine Sahlgrenska Academy, University of Gothenburg, Gothenburg, Sweden

## Abstract

An early pathological hallmark of Alzheimer’s disease (AD) is amyloid-β (Aβ) deposits in the brain, which largely consist of up to 43 amino acids long Aβ peptides derived from the amyloid precursor protein (APP). We previously identified a series of sialylated Tyr-10 O-glycosylated Aβ peptides, 15–20 residues long, from human cerebrospinal fluid (CSF) and observed a relative increase of those in AD vs non-AD patients. We report here on the synthesis and use of an isotopically double-labeled Aβ1-15 glycopeptide, carrying the core 1 Galβ3GalNAcα1-*O*-Tyr-10 structure, to (1) identify by HCD LC-MS/MS the definite glycan core 1 structure of immunopurified and desialylated Aβ glycopeptides in human CSF and to (2) establish a LC-MS/MS quantification method for desialylated Aβ1-15 (and Aβ1-17) glycopeptides and to (3) compare the concentrations of these Aβ glycopeptides in CSF from 20 AD patients and 20 healthy controls. Although we unambiguously identified the core 1 structures and Tyr-10 attachment sites of the glycopeptides, we did not observe any quantitative differences, determined through both peptide and oxonium ion fragments, of the desialylated Aβ1-15 or Aβ1-17 glycopeptides between the AD and non-AD group. The new quantitative glycoproteomic approach described, using double-labeled glycopeptide standards, will undoubtedly facilitate future studies of glycopeptides as clinical biomarkers but should also embrace sialylated Aβ standards to reveal specific sialylation patterns of individual Aβ glycopeptides in AD patients and controls.

## Introduction

The amyloid precursor protein (APP) is a type I transmembrane glycoprotein that undergoes proteolytic processing along two major pathways: the amyloidogenic and the non-amyloidogenic pathway^1^. In the amyloidogenic pathway, two enzymes (β- and γ-secretase) cleave APP into several Aβ peptide variants. The 42-amino acid long Aβ peptide, Aβ1-42, is considered to have a direct link to Alzheimer’s disease (AD) since it is a major constituent of amyloid plaques and has been shown to induce neuropathological changes, such as neuronal and synaptic loss^2^. There is also a range of presumably less toxic Aβ peptides released during APP processing, for instance Aβ1-38, 1-39 and 1-40. In the non-amyloidogenic pathway, APP is cleaved by α-secretase in the middle portion of the Aβ sequence resulting in shorter peptides, which are thought to protect from amyloid deposition in the brain. In general, the proteolytic destiny and lifetime of proteins are not only governed by the availability and specificity of proteases but also by the presence of glycans at specific glycosites, which may affect the cleavage efficiency of nearby proteolytic sites. Such an effect has been reported to be biologically valid for the shedding of other transmembrane glycoproteins where the cleavage site is in close proximity to the membrane^3^. An increasingly important strategy for investigating glycoproteins is to undertake glycoproteomic analyses where glycopeptides, either natively occurring or produced by protease digestions, are structurally characterized by mass spectrometry^4–7^.

To this end, we previously developed a method based on liquid chromatography – tandem mass spectrometry (LC-MS/MS) with electron capture dissociation (ECD) and collision induced dissociation (CID) fragmentation techniques, which enabled the identification of several glycosylation sites of endogenous peptides originating from APP in human cerebrospinal fluid (CSF)^8^. In addition to O-glycosylations at several Ser/Thr residues of APP, we identified a series of glycopeptides uniquely O-glycosylated at Tyr-10 in shorter Aβ peptides, for instance Aβ1-15 and Aβ1-17 (DAEFRHDSGYEVHHQKL). We later expanded this glycosylation to additional mammals by the detection of similar Aβ1-15 glycopeptides in feline CSF^9^. This was the first known example of mammalian Tyr glycosylation of an extracellular protein, but several examples have since then been identified indicating that Tyr glycosylation is widely occurring, although scarce compared to Ser/Thr glycosylations^10,11^. We previously found that the relative concentration of the natively occurring sialylated Tyr O-glycosylated Aβ peptides were increased in CSF samples from AD patients relative to those of non-AD controls (n = 6 + 7), indicating that the presence of a Tyr-10 glycan might have an impact on the metabolic pathway of APP and thus become a candidate biomarker for AD^8^.

The Tyr O-glycosylation of Aβ were mainly composed of NeuAcHex(NeuAc)HexNAc-*O*-Tyr and NeuAcNeuAcHex(NeuAc)HexNAc-*O*-Tyr structures (SA_2_ and SA_3_, see Fig. 2B). For mucin-type O-glycosylation of Ser and Thr, the HexNAc-*O*- core residue is composed of GalNAcα1-*O*-, but given the novelty of the Tyr attachment residue, the precise structure of the HexHexNAc-*O*-Tyr structure was initially uncertain. Therefore, we synthesized GalNAcα1-*O*-Tyr and GalNAcβ1-*O*-Tyr derivatized Aβ1-15 glycopeptides and found that the α-form shared diagnostic fragment similarities in their CID-based MS/MS analyses, thus assigning the native structure to be α-linked^12^, similar to the mucin-type O-glycosylation. Additionally, we presumed that the hexose was a galactose (Gal) since this is the classical hexose found linked to GalNAc and also the only known hexose to which N-acetylneuraminic acid (NeuAc) is linked in mammalian glycoproteins. Recently, we showed that LC-MS/MS with higher-energy collision induced dissociation (HCD) is beneficial to use for the discrimination between GalNAc and GlcNAc containing glycopeptides^13,14^. HCD-MS^2^ spectra were typically recorded over the *m/z* 100–2000 region and in the *m/z* 100–200 mass region. The relative abundances of HexNAc generated oxonium ions were significantly different for GalNAc and GlcNAc containing structures, enabling conclusions about their identities. Moreover, very recently we found that the combined intensities of the HexNAc oxonium ions at *m/z* 204 and *m/z* 366 differed significantly from the combined intensities of the Neu5Ac oxonium ions at *m/z* 274 and *m/z* 292, when Neu5Ac was linked α2,3- or α2,6- to Gal^15^. In the present study we have used these two oxonium ion-based methodologies to assign the core glycan structure of Aβ1-15 and Aβ1-17 *O*-glycopeptides to Galβ3GalNAcα1-*O*-Tyr and the disialylated SA_2_ glycan to Neu5Acα2,3 Galβ3(Neu5Acα2,6)GalNAcα1-*O*-Tyr. Further support for this assignment was accomplished by the synthesis and use of the synthetic core 1 disaccharide Galβ3GalNAcα1-*O*-Tyr substituted Aβ1-15 peptide **1**, which was isotopically labeled on both the peptide and glycan parts. When added to the CSF prior to the Aβ immunopurification (IP) protocol, the compound **1** served as an internal standard for LC-MS/MS based identification and quantitation of desialylated Aβ1-15 and Aβ1-17 *O*-glycopeptides.

The use of isotopically labeled peptide standards in LC-MS/MS methods is an efficient and well-established way to quantitate proteins and peptides in biological samples^16^. However, studies of glycoproteins and glycopeptides are inherently much more challenging due to the macro- and micro-heterogeneity of each conjugate varying in occupancy of glycosites and in glycan structures. Thus, studies including the use of glycoprotein and glycopeptide standards, especially synthetic ones, are scarce, although essential for absolute quantification. It is therefore necessary to develop reference methods aimed at analyzing such standards together with the biological samples. By the use of an Aβ immunopurification protocol^17,18^, LC-MS/MS with HCD fragmentation and an isotopically labeled Aβ1-15 glycopeptide, we have now developed a method to quantify in parallel the absolute concentrations of the Aβ1-15 glycopeptides and peptide in CSF samples. We examined CSF samples from 20 well-characterized AD patients to determine possible alterations of Aβ1-15 (and Aβ1-17) glycopeptide concentrations compared to 20, age and sex matched non-demented, controls.

## Results

### Synthesis of a Tyr-10 glycosylated and isotopically labeled Aβ1-15 glycopeptide

**Figure 1:**
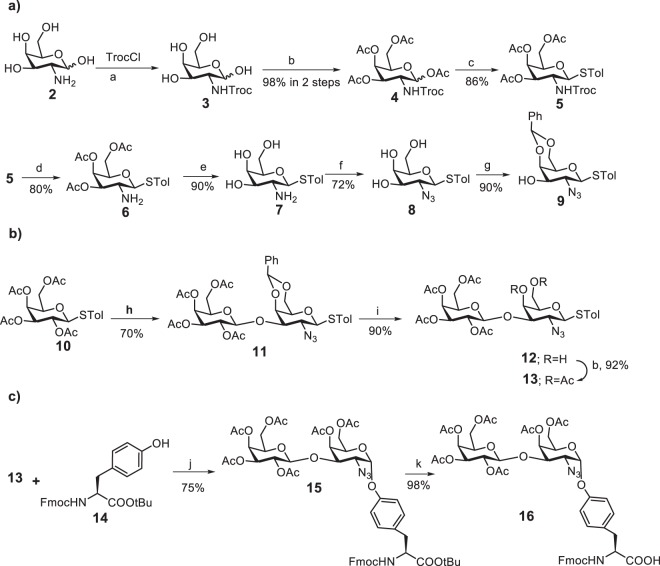
Reagents and conditions: (**a**) NaHCO_3_, H_2_O; (**b**) Ac_2_O, pyridine, DMAP, 0 °C to rt; (**c**) *p*-toluenethiol, DCM, BF_3_.Et_2_O, rt; (**d**) Zn, MeOH:AcOH:DCM (2:1:1); (**e**) NaOMe/MeOH; (**f**) triflic azide, K_2_CO_3_, CuSO_4_, H_2_O/toluene/MeOH, rt; (**g**) benzaldehyde dimethylacetal, *p*-toluenesulfonic acid, acetonitrile; (**h**) molecular sieves (MS) 4 Å, AgOTf, *p*-TolSCl, **9**, TTBP, −78 °C, DCM; (**i**) aq. AcOH (80%), 80 °C; (**j**) 4 Å MS, AgOTf, *p*-TolSCl, TTBP, −78 °C, DCM; (**k**) TFA/ H_2_O (9/1).

Tyrosine glycosylations of peptides and proteins are rare compared to the corresponding serine/threonine glycosylations. As a result, synthesis of the first glycopeptide with Tyr glycosylation was only recently reported^12^ and contained a monosaccharide GalNAc linked to Tyr. Herein, we describe the first synthesis of an istopically double-labeled glycopeptide containing a disaccharide functionalized Tyr residue. In order to prepare the target Tyr-10 glycosylated Aβ glycopeptide **1**, 9-fluorenylmethoxycarbonyl (Fmoc)-protected glycosylated Tyr *tert*-butyl ester **16** was chosen as the key precursor. We started the synthesis of **16** from commercially available D-galactosamine **2**, with its amino group first protected with trichloroethoxycarbonyl (Troc) followed by global acetylation with acetic anhydride (Ac_2_O) to **4** (Fig. 1). Subsequently, the product **4** was allowed to react with 4-methylbenzenethiol in the presence of the Lewis acid boron trifluoride etherate affording predominately *β*-thioglycoside **5**. As the stereochemistry of the glycosidic linkage with Tyr should be *α*-linked, we introduced an azido group to replace the Troc moiety and thereby facilitated the formation of the *α*-glycosyl linkage. The Troc moiety was cleaved with zinc and acetic acid with subsequent removal of the Ac protecting groups (to prevent Ac migration to the free amino group) by treatment with sodium methoxide (NaOMe) in methanol producing **7** in 85% yield over two steps. Compound **7** underwent copper catalyzed diazo transfer reaction^19^ to afford **8** in 72% yield. In order to obtain a glycosyl acceptor suitable for selective galactosylation at the position 3-OH, **8** was converted into the corresponding 4,6-benzylidene acetal **9** by protection of the 4-OH and 6-OH groups in 90% yield using benzaldehyde dimethyl acetal and catalytic amounts of *p*-toluenesulfonic acid in acetonitrile.

To access the Galβ3GalNAc disaccharide, acceptor **9** was coupled with thiogalactoside donor **10** using the pre-activation protocol^20–22^ with the promoter *p*-TolSOTf formed *in situ* through reaction of *p*-TolSCl and AgOTf ^23^, producing the desired *β*-glycoside disaccharide **11** in 70% yield. Acidolytic cleavage of the benzylidene was carried out by treating **11** with 80% aqueous acetic acid at 80 °C to give **12** in which the two OH groups were then protected by reaction with Ac_2_O in pyridine and in presence of dimethylaminopyridine (DMAP) to afford the disaccharide donor **13** in 91% yield over two steps. NMR analysis of **13** (^3^J_H1−H2_ = 7.8 Hz) confirmed that the newly formed glycosidic linkage was *β*. In parallel, tyrosine tert-butyl ester **14** was prepared by reacting Fmoc-Tyr-OH with tert-butanol (tBuOH) in presence of dicyclohexylcarbodiimide (DCC)^24^. Once we had disaccharide donor **13** and tyrosine acceptor **14** in hand, glycosylation was performed using the *p*-TolSOTf promoter^19^ affording the desired glycosylated tyrosine **15** in 75% yield along with 12% of the β anomer separated by silica gel column chromatography. The *α-*glycosidic linkage in **15** was confirmed by NMR analysis (^3^J_H1−H2_ = 2.9 Hz and ^1^J_C−H_ = 180 Hz)^25^. Compound **15** underwent *tert*-butyl ester cleavage by TFA and H_2_O (9:1). This condition did not affect the glycosidic bond and furnished the glycosylated tyrosine building block **16** in a yield of 98%.

The assembly of Aβ glycopeptides was carried out starting from commercially available 2-chlorotrityl resin loaded with glutamine (Supplementary Fig. 1) using Fmoc-based solid phase peptide synthesis^26^. In each coupling cycle, Fmoc-amino acid (5 eq.) was activated by *O*-(1*H*-benzotriazol-1-yl)-*N*,*N*,*N*′,*N*′-tetramethyluronium hexafluorophosphate (HBTU) (5 eq.), 1-hydroxybenzotriazole (HOBt) (5 eq.) and Hünig’s base (*N*,*N*-diisopropylethylamine, DIPEA) (10 eq.). After each coupling, unreacted amino groups were capped with Ac_2_O, and then Fmoc groups were removed with piperidine in DMF (20%). For the addition of glycosylated tyrosine building block **16**, *O*-(7-azabenzotriazol-1-yl)-*N*,*N*,*N*′,*N*′-tetramethyluronium hexafluorophosphate (HATU), 1-hydroxy-7-azabenzotriazole (HOAt) and DIPEA were utilized as coupling agents^27,28^.

**Figure 2:**
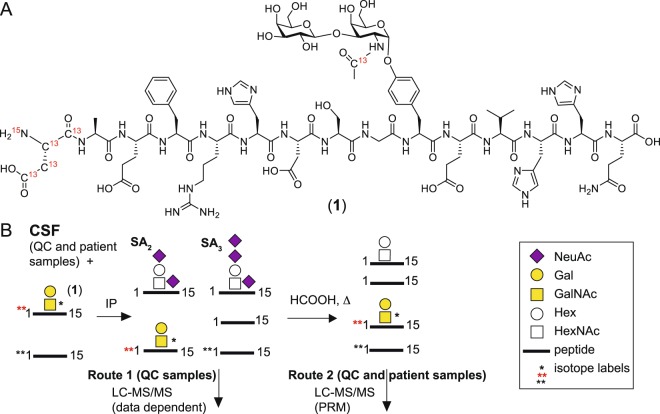
Structure of Aβ1-15 glycopeptide standard 1 and schematic protocol for the Aβ immunopurification and LC-MS/MS of CSF samples. (**A**) Structure of Galβ3GalNAcα1-*O*-Tyr glycosylated Aβ1-15 having ^15^N and ^13^C isotope labels on Asp-1 and ^13^C on the GalNAc *N*-acetyl group. (**B**) The **1** and Aβ1-15** internal standards were added to CSF samples and co-immunopurified using antibody 6E10 coated magnetic beads. The co-immunopurified internal standards as well as native Aβ peptides and glycopeptides, from QC samples, were directly subjected to LC-MS/MS (Route 1) or to acid hydrolysis of sialic acid residues, QC and patient samples, and then analyzed with the PRM assay (Route 2). A range of Aβ peptides and glycopeptides were co-immunopurified, but only Aβ1-15, glycosylated Aβ1-15, Aβ1-17, and glycosylated Aβ1-17 were included in the PRM assay.

Upon completion of glycopeptide chain assembly on solid phase, the glycopeptide **17** was obtained in 19% overall yield from the resin by cleavage with a mixture of TFA: TIPS: water (95:2.5:2.5). Conversion of the azido group of the glycopeptide **17** into an acetamido group was first tested by treating the glycopeptide with activated zinc in Ac_2_O and acetic acid, which unfortunately led to the decomposition of the glycopeptide. The azide in **17** was successfully reduced to amine with zinc in MeOH:AcOH:DCM (2:1:1). The resulting amine was then acetylated with Ac_2_O in MeOH generating the *N*-acetamido glycopeptide **19** in 91% yield over two steps (Supplementary Fig. 1). The final glycopeptide **20** was produced in 95% yield by global deprotection of Ac and Fmoc groups with sodium methoxide in methanol. In the same manner as for glycopeptide **20**, glycopeptide **1** was synthesized (Fig. 2A) with the exception that ^13^C_4_, ^15^N labeled Fmoc-Asp(OtBu)-OH building block and ^13^C labeled Ac_2_O were utilized to introduce the isotopic labels corresponding to +5 u at Asp-1 and +1 u at the glycan to aid in mass spectrometric quantification studies. This is the first time such a glycopeptide standard bearing a disaccharide on the Tyr moiety has been synthesized. The synthetic approach developed can be applied to Tyr glycosylated peptides in general.

### Immunopurification and LC-MS/MS analysis of native Aβ glycopeptides from CSF

**Figure 3:**
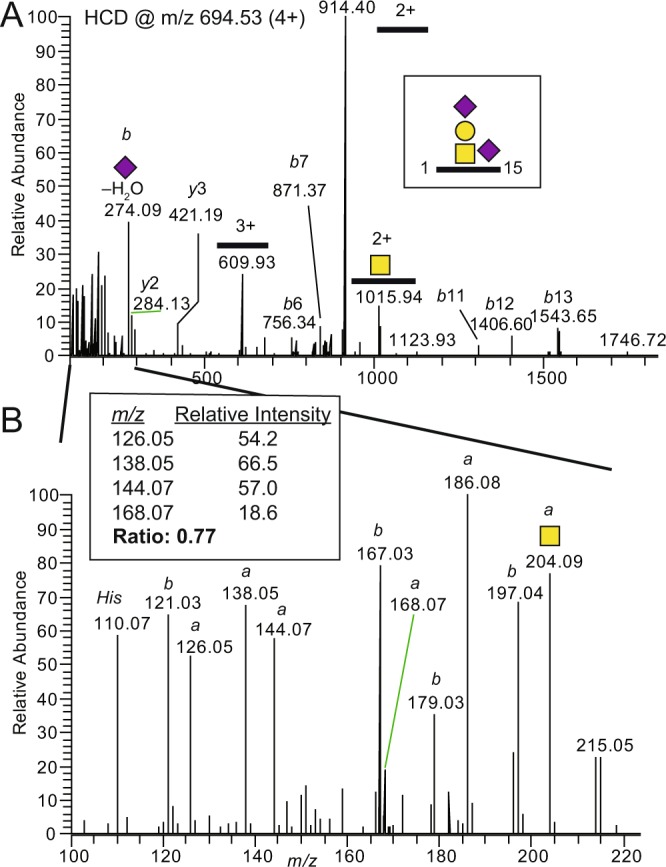
HCD-MS^2^ of immunopurified SA_2_-Aβ1-15 from CSF samples. (**A**) Full range MS^2^ spectrum of SA_2_-Aβ1-15 glycopeptide immunopurified from pooled CSF (Route 1, Fig. 2B) demonstrating glycosidic and peptide backbone fragmentation and (**B**) expansion of the *m/z* 100–220 region showing typical oxonium ions. Oxonium ions originating from the HexNAc are marked *a* and oxonium ions originating from the Neu5Ac are marked *b*. The relative intensities of the HexNAc ions and the calculated “GlcNAc/GalNAc” ratio are boxed. *His* is from the histidine side chain immonium ion.

To develop a robust LC-MS/MS-based method for the quantification of Aβ1-15 glycopeptides, CSF quality control (QC) samples were spiked with **1** and isotopically labeled Aβ1-15 peptide (Aβ1-15**) to be used as internal standards. Anti-Aβ antibody clone 6E10 was conjugated to magnetic protein G beads for the immunopurification of Aβ peptides and glycopeptides^17^ and the eluted samples were analyzed with nanoflow LC-MS/MS (Fig. 2B, Route 1). Native Aβ peptides and glycopeptides were identified by Mascot database searches (Supplementary Table 1) as well as by manual analysis and included ~30 Aβ peptide isoforms, most of which started at the Aβ Asp-1 residue (Asp-672 of Uniprot ID P05067) but also some that started at residues 3, 4, 5 and −25 in relation to Asp-1. The HCD-MS^2^ spectrum of the native disialylated HexHexNAc substituted Aβ1-15 glycopeptide (SA_2_-Aβ1-15) (Fig. 3A) demonstrated the presence of several diagnostic oxonium ions at *m/z* 126, 138, 144, 168, 186, 204 (Fig. 3B), and *m/z* 274 (Fig. 3A). We have previously shown that the summed intensities of the peaks at *m/z* 138 and *m/z* 168 divided by the summed intensities at *m/z* 126 and *m/z* 144, i.e., (138 + 168)/(126 + 144) intensity ratio, also named GlcNAc/GalNAc ratio, is 0.5–1.5 for Galβ3GalNAc and 2–20 for Galβ4GlcNAc structures, thereby discriminating these two structures^13^. For SA_2_-Aβ1-15 the ratio was 0.77, demonstrating that the Tyr glycosylation of Aβ peptides is indeed composed of the core 1 Galβ3GalNAcα1-*O*- structure and is identical to the mucin *O*-glycosylation of Ser/Thr residues. Additional support for the Galβ3GalNAc structure was obtained from oxonium ion fragment analysis of the natively occurring SA_2_-Aβ4-15, SA_2_-Aβ1-16, SA_2_-Aβ1-17, and SA_2_-Aβ1-19 glycopeptides, which all had GlcNAc/GalNAc ratios of 0.74–0.85 (Supplementary Fig. S2). As previously, we could not detect any glycosylation of the longer Aβ1-38, Aβ1-39, Aβ1-40, and Aβ1-42 peptides. Furthermore, additional glycoforms of Aβ1-15 glycopeptides having SA_1_, SA_3_ and *O*-acetylation of SA_3_ (SA_3_-*O*Ac) structures were identified, and all had GlcNAc/GalNAc ratios of 0.62-1.11 showing that these sialic acid glycoforms did not significantly perturb the GlcNAc/GalNAc diagnostic ratios, and all were in support of the core 1 Galβ3GalNAc-*O*- structure (Supplementary, Fig. S3).

### Design of the quantitative analysis

Compound **1** and Aβ1-15** peptide were designed and used as internal standards for quantification of Aβ1-15 glycopeptides and peptides, respectively, and were added to the CSF samples immediately before any work-up steps. The standards had the correct and identical peptide sequence, including the sequence reactive towards the 6E10 antibody, but compound **1** also contained the unique double labeled Tyr10-*O*-glycan core 1 structure. Since we did not detect this non-sialylated core 1 disaccharide structure as a native structure in CSF, we undertook the strategy to desialylate the endogenous glycopeptides by hydrolyzing the IP sample with formic acid at 80 °C^29^. Thus, all glycoforms of the endogenously sialylated Aβ1-15 glycopeptides were transformed into the Galβ3GalNAc-Aβ1-15 desialylated glycopeptide, reducing the heterogeneity and increasing the precursor signal intensity (Fig. 2B, Route 2). Furthermore, the chromatographic mobility and the ionization efficiency should now be practically identical for endogenous Galβ3GalNAc-Aβ1-15 and the isotopically labeled **1**, enabling their simultaneous fragmentation and differentiation of native and isotopically labeled fragment ions. The HCD-MS^2^ spectrum of the pure standard compound **1** showed that the LC-MS/MS measured relative abundance of the residual non-labeled oxonium ions at *m/z* 138.06, 168.07, 186.07, and 204.08 were at the ~2% level compared to their +1 *m/z* counterparts and the heavy isotope incorporation was thus 98% for the glycan part (Supplementary Fig. S4C). Additionally, the precursor ions peaks at *m/z* 731.64 (non-labeled peptide, labeled glycan) and *m/z* 732.98 (labeled peptide, non-labeled glycan) had only 1% and 3%, respectively of the signal intensity of the fully labeled **1** (Supplementary Fig. S4A). For Aβ1-15** no precursor ion or b-ions corresponding to the non-labeled peptide (−10 u) were detected for IP samples where the CSF was excluded. These low amounts of light isotope residuals on both the peptide and glycan parts made direct measurements of relative abundances using either b-ions or oxonium ions possible.

### Optimization of the pre-analytical procedure

**Figure 4:**
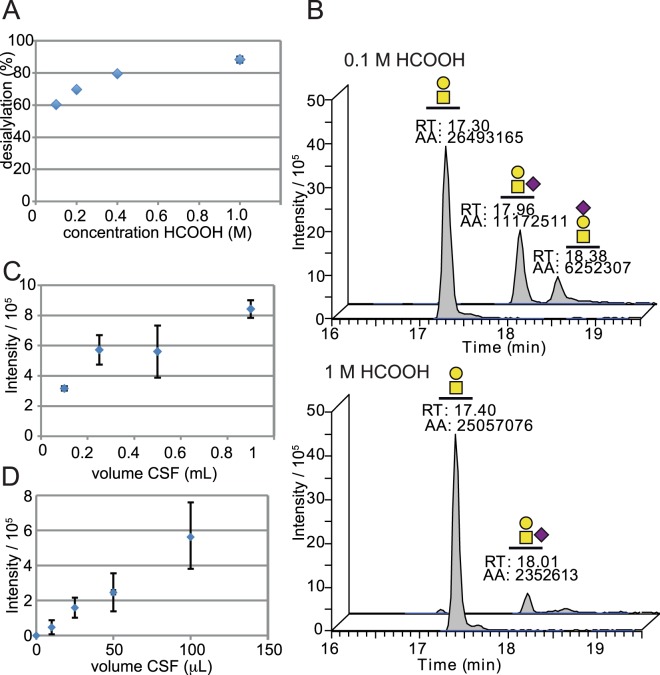
Desialylation and sample volume optimization. (**A**) Desialylation efficiency of endogenous Aβ1-15 glycopeptides at different concentrations of formic acid. (**B**) Extracted ion chromatograms of Galβ3GalNAc-Aβ1-15 and SA_1_-Aβ1-15 glycopeptides after 0.1 M (top) and 1.0 M formic acid sialic acid hydrolysis (bottom). (**C**) CSF volume titration showing precursor ion signal intensity versus CSF sample volume of 100–1000 μL, and (**D**) 0–100 μL CSF from two separate experiments.

Initially we used 0.1 M formic acid for the desialylation step (Fig. 2B, route 2), but the degree of desialylation was then just 60% (Fig. 4A). These results are in accordance with the literature where 0.5 M formic acid was shown to result in 60–80% sialic acid removal from glycoproteins^30^. By stepwise increasing formic acid concentrations from 0.1 M to 1.0 M, we found that 1.0 M was required to remove the sialic acids by >90% (Fig. 4A) and still did not result in any measurable glycopeptide degradation since the precursor ion intensities were maintained intact (Fig. 4B, top and bottom chromatograms). The two partially hydrolyzed SA_1_ glycoforms, one having Neu5Ac added α2,6 to the GalNAc and the other having Neu5Ac added α2,3 to Gal, were chromatographically well separated (Fig. 4B) and were attributed to their respective elution times by analysis of their GalGalNAc and Neu5Ac generated oxonium ion ratios (Supplementary Fig. S5)^15^.

In order to gain access to valuable CSF samples from AD patients and controls, we also had to minimize the CSF sample volume compared to our earlier studies. We found that, by varying the amount assayed from 10 μL to 1 mL of QC CSF, it was possible to get sufficient spectral intensities from 25 μL to 1 mL of CSF and we settled for the use of 100 μL CSF to accommodate samples even with low Aβ glycopeptide concentrations (Fig. 4C,D).

### Quantification by parallel reaction monitoring of Galβ3GalNAc-Aβ1-15 and 1 in CSF

**Figure 5:**
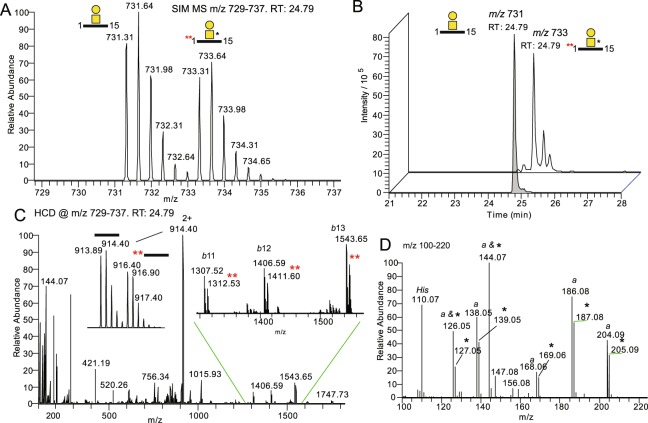
PRM MS^2^ of desialylated Aβ1-15 glycopeptides and standard 1. (**A**) A SIM acquisition of the co-eluting precursors Galβ3GalNAc-Aβ1-15 (*m/z* 731.31) and **1** (*m/z* 733.31), corresponding to Route 2 (Fig. 2B) using QC CSF, and (**B**) their extracted ion chromatograms. (**C**) Full range MS^2^ spectrum of the two precursor ions. The inserts show *m/z* expansion of the peptide ions and b-ions demonstrating the 5 u mass difference between the peptide b-fragments of Galβ3GalNAc-Aβ1-15 and **1**. (**D**) Expansion of the *m/z* 100–220 region showing the oxonium ions from the native glycopeptide marked *a* and the isotope labeled ions from **1** are marked *.

Formic acid treated samples were subjected to LC-MS/MS where both single ion monitoring (SIM) and parallel reaction monitoring (PRM) acquisitions were collected. The [M + 3 H]^3+^ precursor ions of the endogenous Galβ3GalNAc-Aβ1-15 and **1** co-eluted (Fig. 5A,B), supporting the interpretation of the fragment analyses that they have indeed identical configurations. The HCD spectrum, using an *m/z* 729–737 isolation window encompassing the masses of both precursors (Fig. 5C), showed fragmentation peaks originating from both light and heavy precursors. The glycosidically fragmented 2+ peptide ions (Y_0_ ions) at *m/z* 914.40 and *m/z* 916.40 (Fig. 5C, left insert) and peptide backbone fragmented *b*-ions (Fig. 5C, right insert) all had the additional mass of 5 u corresponding to the labeled Asp-1 residue (Fig. 2A). The light/heavy relative intensities equated directly to their relative concentrations assuming an equal binding constant of the 6E10 antibody for the endogenous sialylated glycoforms and for the non-sialylated glycopeptide **1** as well as a quantitative desialylation step for the endogenous glycopeptides.

Since also the GalNAc of **1** was isotopically labeled, the ratios between light and heavy oxonium ions, at the *m/z* 100–220 interval, were additionally used for relative quantifications of the Aβ1-15 glycopeptides (Fig. 5D). Here, the ions annotated with * are from **1** and those from the native Galβ3GalNAc-Aβ1-15 are again marked with *a* (compare Fig. 3B). HCD-MS^2^ spectra of the individual precursor ions (*m/z* 731.31 and 733.31, Supplementary Fig. 6) demonstrated that the oxonium ions at *m/z* 144, and partly at *m/z* 126, were both due to loss of the acetyl group, which includes loss of ^13^C from **1**. This is in full accordance with our previous study describing the HCD induced decomposition of HexNAc-generated oxonium ions^14^. The fragment ions at *m/z* 144 and *m/z* 126 should thus be omitted from quantification analyses using isotopic tags situated on the HexNAc *N*-acetyl group. Instead, only the ion pairs at *m/z* 138/139, *m/z* 168/169, *m/z* 186/187 and *m/z* 204/205 were used.

### Quantitative analysis of Aβ1-15 glycopeptide in CSF samples from AD patients and healthy controls

**Figure 6:**
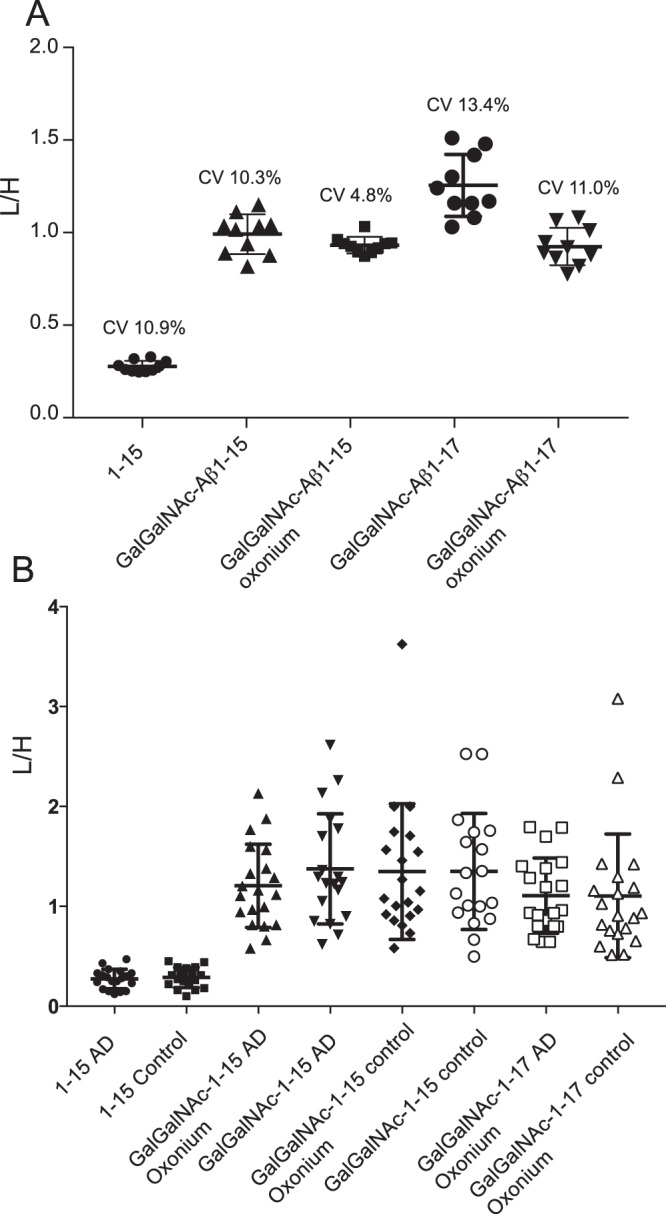
Relative quantification of Aβ glycopeptides and peptides. (**A**) Quantification, L/H ratios, of Aβ1-15 (peptide fragments), of Galβ3GalNAcα-Aβ1-15 (peptide fragments and oxonium ions), and of Galβ3GalNAcα-Aβ1-17 (peptide fragments and oxonium ions) from 10 QC CSF samples analyzed together with the 40 patient samples. (**B**) Quantification of Aβ1-15, Galβ3GalNAcα-Aβ1-15 and Galβ3GalNAcα-Aβ1-17 using the same ratios as above for CSF samples of 20 AD patients and 20 matched healthy controls. The simultaneously measured ratios of Aβ1-17 peptide versus Aβ1-15** for the QC, AD and control samples are shown in Supplementary Fig. 7A.

We finally assayed CSF samples from 20 individuals with AD (9 men) and 20 non-demented controls (9 men), to assess potential differences in concentrations of Aβ1-15 glycopeptides between the two groups. The mean (SD) age of the AD patients and controls were 64 (5) and 61 (7) years, respectively; and mean years of education were 13 years for both groups. The *APOE* genotype, CSF/serum albumin ratio, and core AD CSF biomarker (P-tau, T-tau and Aβ1-42) levels for each individual are provided in Supplementary Table 2. Ten QC samples were assayed amid the 40 patient samples during the experimental procedures. The total coefficient of variation (CV) of the light-to-heavy (L/H) ion signal ratio for Galβ3GalNAc-Aβ1-15 and **1** for the 10 QC samples was 10.3%, and L/H for Aβ1-15 and Aβ1-15** was 10.9% (Fig. 6A and Supplementary Table 2). The CV for the L/H of Galβ3GalNAc-Aβ1-17 versus **1** was 13.4% and L/H for Aβ1-17 versus Aβ1-15** was 16.3% (Supplementary Fig. 7A). Furthermore, the isotope labeled oxonium ions of **1** and the non-labeled ones from co-eluting Galβ3GalNAc-Aβ1-15 and from Galβ3GalNAc-Aβ1-17 were used to calculate their relative concentrations, which gave similar relative ratios as when using peptide backbone fragment analysis with CV’s of 4.8% and 11.0%, respectively (Fig. 6A). As a comparison, absolute intensity signals from the endogenous glycopeptides and the internal standards provided CVs of 24.5–29.8% based on the peptide backbone fragments and 21.9–23.1% based on the oxonium ions (Supplementary Table 2), demonstrating clearly that the L/H measurements provided more stable and thus more reliable results.

For the 40 AD and non-demented control samples, the Aβ1-15 peptide L/H isotope ratios were similar for the two groups (Fig. 6B). This was also the case for the Aβ1-17-to-Aβ1-15** ratio (Supplementary Fig. 7A) and both results are in accordance with previous studies^31^. Further, for endogenous Galβ3GalNAc-Aβ1-15 versus **1** there were no differences between the two groups as measured either using the peptide fragment ion- or the oxonium ion methodology. In addition, Galβ3GalNAc-Aβ1-17 versus **1**, based on both the peptide and the oxonium ions, was measured and was also found similar between the two groups (Fig. 6B and Supplementary Table 2). Thus, we could not detect any differences of the enriched desialylated Aβ glycopeptide concentrations between the AD and non-demented controls using this sample set and methodology. Importantly, 9 out of the 20 AD patient samples, and 8 out of the 20 non-AD control samples had an AD biomarker profile of P-tau, T-tau and Aβ1-42, which did not fit with the clinical diagnosis (Supplementary Table 2). However, when these samples were removed from the Aβ ratio plots, still no changes were found between the two groups (Supplementary Fig. 7B). Significantly, the peptide fragment ion analysis and oxonium ion analysis gave the same results showing that it is straightforward to use, not only peptide fragments, but also oxonium ions for quantitative analyses of standard glycopeptides isotopically labeled on the glycan part.

## Discussion

Methodological advances in the field of LC-MS/MS identification of glycopeptides have excelled during the last ten years^4–7,32^ and these glycoproteomic approaches have partly been possible due to the development of faster, more versatile and more stable LC-MS/MS instrumentation. In particular, HCD has become an important fragmentation technique to provide fast and sensitive glycopeptide fragmentation analysis in one MS^2^ step. For HCD-MS^2^ spectra, the full *m/z* 100–2000 mass region can be covered, and in the *m/z* 100–400 interval, diagnostic oxonium ions originating from *N*-acetyl containing saccharides dominate the fragment spectra (Fig. 3). The presence of such oxonium ions has become an important tool in the fragment analysis of glycopeptides^4,33,34^. Also, intensity ratios of the HexNAc specific oxonium ions, incorporated into a GlcNAc/GalNAc ratio, have been introduced to identify glycopeptides having GlcNAc or GalNAc structures^13,35,36^. The application of this technique has become increasingly important, specifically since a recent study identified extended extracellular *O*-GlcNAc glycosylation as type 2 chain Galβ4GlcNAc-*O*-Ser/Thr, additionally modified with a Neu5Ac residue linked to the Gal^37^. Previously, core 1 mucin glycosylation, i.e. Galβ3GalNAcα1-*O*-Ser/Thr was the only anticipated HexHexNAc modification of Ser/Thr. When we compared MS^2^ spectra of CID fragmented GalNAcα1-*O*-Tyr and GalNAcβ1-*O*-Tyr standards with native Aβ1-15 glycopeptides the stereochemistry of the endogenous glycopeptide was settled to be in an α configuration^12^. However, due to the uniqueness of the HexHexNAc-*O*-Tyr glycosylation of short Aβ peptides, it was not obvious whether the glycan was composed of Galβ3GalNAcα−, Galβ4GlcNAcα−, or something else. However, initiation with GalNAcα-*O*-Tyr was more probable since this structure was also identified on >40 Tyr sites using a Simple cell technology, which is a cell-based methodology where GalNAcα1-*O*- is formed but its elongation inhibited^10,38^.

In this study we show by HCD fragmentation and comparisons with the synthetic Galβ3GalNAcα-Aβ1-15 standard **1** that the Aβ glycopeptides of human CSF indeed carry a core 1 Galβ3GalNAcα1-*O*-Tyr structure. The precise identity of the Galβ3GalNAcα1-*O*-Tyr structure was verified by the simultaneous PRM LC-MS/MS analysis of co-eluting **1** and native HexHexNAc-Aβ1-15 glycopeptides. Since the compound **1** glycopeptide was isotopically labeled on Asp-1 and at the *N*-acetyl part of GalNAc, both the peptide backbone b-ions and the oxonium ions differed in mass due to isotope labels and were used to compare their fragmentation profiles (Fig. 5). In order to directly compare the fragmentation pattern of endogenous versus added **1**, the sialic acid residues were removed to >90% by treatment with 1 M formic acid at 80 °C (Fig. 4). At lower acid concentrations, partial release of one Neu5Ac from the Gal or GalNAc of SA_2_ into two forms of SA_1_ was used for MS^2^ fragment analysis and identification of their isomeric structures.

We recently showed that the sialic acid α2,3/α2,6 isomeric modification of glycopeptides could be distinguished by analysis of the intensity ratios of the HexHexNAc *m/z* 204 + *m/z* 366 ions versus the Neu5Ac *m/z* 274 + *m/z* 292 oxonium ions^15^. An oxonium ion analysis of these ions from the partially hydrolyzed SA_2_-Aβ1-15 glycopeptide (Supplementary Fig. 5) showed clearly that it had a NeuAcα2,3 Galβ3(Neu5Acα2,6)GalNAcα1-*O*-Tyr structure. In summary, two separate LC-MS/MS oxonium ion ratios and the use of the carefully prepared Aβ glycopeptide standard **1** were used to assign the detailed structure of the uniquely *O*-Tyr glycosylated Aβ peptides.

The use of isotopically-labeled peptide standards is a well established method for quantification of peptides/proteins in proteomics^16^. However, in the field of glycoproteomics, the use of isotopically-labeled glycopeptide standards, has not been established partly due to the fact that glycoproteomics is a more recently emerging field and partly due to the challenges in synthesizing such labeled standards matching the correct glycan with the correct attachment site in the correct peptide; in this case Galβ3GalNAcα1-*O*-Tyr 10 in Aβ1-15. Here we have employed a general strategy, by which a per-*O*-Acetyl Galβ3GalN_3_α1-*O*-Tyr building block was prepared first (Fig. 1), which was then used as a molecular cassette to incorporate the glycosylated Tyr into the peptide chain. Only at a later stage was the ^13^C isotope introduced (Fig. 1 and Supplementary Fig. 1) facilitating the use of a small amount of ^13^C-acetic anhydride and enabling alternative N-acyl labels to be introduced in the glycopeptide product. This is, to the best of our knowledge, the first time that a glycopeptide standard with a glycan longer than a monosaccharide linked to Tyr has been prepared. This synthetic strategy can be generally applicable to the synthesis of a wide range of Tyr glycosylated peptides.

To aid in the quantification studies, we adapted a unique double labeling strategy. The ^13^C isotope labels were introduced into the glycan, while the peptide was labeled with a more conventional ^13^C and ^15^N labeling of the N-terminal Asp of the glycopeptide Aβ1-15, creating for the first time a double labeled glycopeptide standard for MS quantification analysis. This double labeling enabled an exact compositional tracking and quantification of Galβ3GalNAcα1-*O*-Tyr10 Aβ1-15 glycopeptides in our samples.

The glycopeptide 1 was conveniently used as an internal standard to measure the relative abundances of Aβ glycopeptides in 100 μL CSF samples with our PRM assay. To our knowledge, this is the first example where a fully synthetic glycopeptide standard is used for quantification purposes^32^. The isotope labeling of both the peptide and glycan parts facilitated the analysis based both on the peptide backbone fragment ions and on the GalNAc derived oxonium ions, and both strategies gave similar results (Fig. 6). Quantitative assays based on the relative ratios of endogenous versus isotopically labeled oxonium ions are particularly attractive to use for glycopeptides since the same oxonium ion intensities can be used for different glycopeptides, varying in peptide lengths but having identical glycan modifications, as exemplified here in the simultaneous PRM quantification of the Aβ1-15 and Aβ1-17 glycopeptides.

We analyzed well-defined CSF samples from AD patients and non-demented controls (n = 20 + 20), but did not detect any quantitative differences between the core 1 Tyr-10 glycosylated Aβ1-15 and Aβ1-17 glycopeptides in the two sample sets. This finding is seemingly in contrast to our previous report where increased levels of Aβ glycopeptides were found in AD patients versus non-AD patients (n = 6 + 7). In our original study, we identified and assayed for relative amounts of each and all SA_2_ and SA_3_ Tyr10-glycosylated Aβ glycopeptides in CSF without the use of an internal standard. The LC-MS quantification was based solely on the relative intensities of the respective parent ions and normalized towards the sum of all other parent ions of both Aβ peptides (except for Aβ1-42) and Aβ glycopeptides in each of the 1–5 mL CSF samples analyzed. The amounts of each sample necessary for such an analysis were, however, too large for clinical studies. Thus, in order to increase the analytical sensitivity and reproducibility of the assay we decided to keep the immunopurification but introduced (1) a desialylation step to decrease the glycan heterogeneity and thus increase the concentrations of hydrolyzed, now non-sialylated, core 1 modified Aβ glycopeptides, (2) introduce a double-labeled internal Aβ1−15 glycopeptide standard (**1**) and a single-labeled Aβ1−15 peptide internal standard. The internal standards enabled quantification of both glycopeptides and peptides and a comparison between all samples (Figs 5 and 6B) also giving a measure of the analytical robustness of our PRM method (Fig. 6A). By using internal standards we lowered the CV from 25–30%, when using the peak areas only, to 5–11% both for Aβ1−15 glycopeptide standard (**1**) and for the Aβ1-15. peptide standard. The use of an IS will compensate for several variations during sample preparation and measurement, e.g. immunopurification efficiency, injection volume, chromatographic variations, electrospray variations and ion suppression. In general, the use of an IS for quantitative purposes is also necessary since a C18 desalting step must often be performed prior to LC-MS which, in our experience may result in recovery yield differences of 30%.

One explanation for the previously detected differences of Aβ glycopeptides in AD versus non-AD samples may be due to random sample variations in the relatively small sample sets (n = 13) used. Based on the relative change of 1.5 of the relative signal intensities of each Aβ glycopeptide and less than 30% CV between samples within each group a power analysis gave that 20 + 20 samples would be sufficient to use in order to verify the original results. However, since we did not observe any quantitative differences with the improved method used here, it must be concluded that, based on the used patient sample criteria, there are no quantitative differences in Tyr-10 core 1 O-glycosylation between the AD and non-AD individuals. However, quantitative differences between the degree of sialylation, lactone formation or O-acetylation of NeuAc residues in Aβ glycopeptides, identified in our original study, were not possible to observe in the present study and will need the synthesis of labelled internal standards corresponding to each unique glycan and peptide structure. Regardless of these limitations, the presented use of LC-MS/MS and fragment ion characteristics in combination with isotope labeled glycopeptides illustrates how this approach can be applied for investigating potential differences in concentration of disease-related glycopeptides in future clinical studies.

## Materials and Methods

### CSF samples

Pooled CSF samples were made up from de-identified remaining aliquots from clinical routine analyses. After one freezing cycle, samples were thawed for 1 hour to obtain room temperature, pooled, and then aliquoted and stored at −80 °C. This procedure is approved by the Ethics Committee at University of Gothenburg (EPN 140811). These aliquots were used as quality control (QC) samples in the study, but also for the method development and structural studies. For the clinical study, CSF samples were obtained from 20 AD patients [9 men; mean (SD) age: 64 (5) years] and 20 healthy non-AD control individuals [9 men; 61 (7) years]. The AD patients were obtained from the memory clinic at the Sahlgrenska University Hospital, Mölndal. Informed consent was obtained from all patients and controls of the study. AD was diagnosed according to the NINCDS-ADRDA criteria^39^. The clinical procedures have been described previously^40^. The AD diagnoses were evaluated by two independent specialized physicians that were blinded to the results of CSF biomarkers, but had full access to other clinical data. More specifically, an AD diagnosis was established if the patient fulfilled general dementia criteria and in addition had no or mild white matter changes on magnetic resonance imaging and predominant parietotemporal lobe symptoms, i.e. episodic memory loss and difficulties in interpreting sensory information. If the two evaluators had different opinions, the patient was discussed until a consensus decision could be made in terms of the diagnosis. Healthy controls were recruited through senior citizen organizations, e.g., at information meetings on dementia, and a small proportion are relatives of patients. To be regarded as healthy, the controls should not experience or exhibit any cognitive decline at the time of inclusion in the study. The study was approved by the ethical committee at the University of Gothenburg. The study was conducted according to the Declaration of Helsinki. To estimate the required number of CSF samples to be used, a power analysis was performed via the web-tool at the University of British Columbia homepage (https://www.stat.ubc.ca/~rollin/stats/ssize/n2.html).

### Synthesis of labeled Aβ glycopeptide 1

The synthesis and analysis of glycan building blocks and the solid phase peptide synthesis are presented in the Supplementary experimental procedures. All solvents that were used for moisture sensitive reactions were dried according to standard procedures. All starting materials, solvents and reagents obtained from commercial suppliers were used without further purification. Most of the amino acids were purchased from Chem-Impex, Fmoc-Asp(OtBu)-OH-^13^C_4_,^15^N 98 atom % ^13^C, 98 atom % ^15^N, 97% and ^13^C labeled acetic anhydride were purchased from Sigma-Aldrich. Glutamine(Trt)-2-chlorotrityl resin was from ChemImpex. All other solvent and reagents were purchased from Sigma-Aldrich. Reactions were monitored by thin-layer chromatography with pre-coated silica gel 60 F254 glass plates (Millipore). Flash column chromatography was performed on silica gel 60 (230–400 mesh). ^1^H, ^13^C NMR and and 2D NMR spectra were recorded on an Agilent-500 MHz spectrometer and ^1^H NMR spectra for glycopeptide 1 were recorded on a 900 MHz spectrometer. Peptide purification was performed on a Shimadzu HPLC (LC-8A Liquid Chromatograph Pump, DGU-14A Degasser and SPD-10A UV-Vis Detector) with a SUPELCOSILTM LC-18 HPLC column (length 25 cm×, i.d. 10 mm) and (length 25 cm×, i.d. 4.6 mm). Mass spectra were obtained by ESI mass spectra (Waters Xevo G2-S Q-TOF LC-MS instrument) in positive ion mode and MALDI Q-TOF. Synthetic Aβ1-15 peptide labeled with ^13^C and ^15^N at Arg-5, (Aβ1-15**) was from Thermo Fisher Scientific (purity >97%).

### Characterization of anomeric stereochemistry of glycan building blocks

The stereochemistry of the glycosidic linkages was determined by ^3^*J*_(H1,H2)_ through ^1^H-NMR and/or ^1^*J*_C1,H1_ through gHMQC 2-D NMR (without ^1^H decoupling). The smaller coupling constants of ^3^*J*_(H1,H2)_ (around 3 Hz) for the galactosides indicate *α* linkages and larger coupling constants ^3^*J*_(H1,H2)_ (7.2 Hz or larger) indicate *β* linkages. For all glycosyl linkages, the stereochemistry were further confirmed via ^1^*J*_(C1,H1)_ (around 170 Hz) suggesting *α-*linkages and ^1^*J*_(C1,H1)_ (around 160 Hz) for *β*-linkages^25^.

### Immunopurification protocol

Centrifuged CSF samples, 10–1,000 μL, were diluted with phosphate buffered saline (PBS) to a volume of 1.0 mL. Aβ1-15** (0.1 fmol/μL CSF used) as well as labeled compound **1** (0.3 fmol/μL CSF used) were added to each sample and were agitated at room temperature for 30 min. Protein G magnetic beads (Dynabeads, Thermo Fisher), 50 μL/sample in a 15 mL Falcon tube, were washed three times with 5 mL PBS, and resuspended in 50 μL PBS per used CSF sample. The clone 6E10 Aβ antibody (1 μg/μL, Biolegend Inc., San Diego, CA, USA), 4 μL/sample, was added and the beads were again agitated at room temperature for 1 h. The beads were then washed twice with 5 mL PBS, suspended in 5 mL 20 mM dimethylpimelimidate dihydrochloride (DMP, Sigma Aldrich) in 0.2 M triethanolamine (Sigma Aldrich), and cross-linked under agitation for 30 min. The DMP solution was removed and remains were quenched by addition of 5 mL 50 mM 2-amino-2-(hydroxymethyl)propane-1,3-diol (Tris) buffer and agitated for another 15 min. The beads were then washed twice with 5 mL PBS, 5 mL 0.1% bovine serum albumin (BSA) was added and agitated for 1 h, and then washed once with 5 mL PBS and suspended in 50 μL PBS per CSF sample.

To each CSF sample, 10 μL 2.5% Tween 20 and 50 μL beads were added, and were agitated at 4 °C overnight. The samples were washed and Aβ released by the use of a magnetic bead sorting instrument (Kingfisher, Thermo Scientific). The beads were sequentially washed with 1 mL 0.025% Tween 20 in PBS, 1 mL PBS, 1 mL 50 mM ammonium bicarbonate, and agitated 30 s for each solute, and then bound material was released by treatment with 200 μL of 0.5% formic acid for 4 min. The samples were transferred to microcentrifuge tubes (Costar), lyophilized and stored at −20 °C prior to analysis.

### LC-MS/MS setup

LC-MS/MS was performed by nanoflow LC (Dionex Ultimate 3000 system, Thermo Fisher Scientific) coupled to ESI quadrupole–orbitrap MS and MS/MS (Q Exactive equipped with a Nanospray Flex ion source, Thermo Fisher Scientific). Chromatography was performed with a reversed-phase Acclaim PepMap C18 (length 20 mm, i.d. 75 µm, particle size 3 µm, pore size 100 Å) trap column used for online desalting and sample clean-up, followed by a reverse-phase Acclaim PepMap RSLC C18 (length 150 mm, i.d. 75 µm, particle size 2 µm, pore size 100 Å, both Thermo Fisher Scientific) column. Separation was performed at a flow rate of 300 nL/min by applying a linear gradient of 5–40% B for 50 minutes for exploratory work or 5–20% B for 20 minutes for quantitative analysis using parallel reaction monitoring (PRM). Mobile phase A was 0.1% formic acid in water (v/v) and mobile phase B was 0.1% formic acid/84% acetonitrile in water (v/v/v).

For explorative work mass spectra were acquired in positive ion mode with a voltage setting of +1.7 kV and a resolution setting of 70,000. Target values were 1 × 10^6^ and maximum isolation time 250 ms both for MS and MS/MS acquisitions. MS acquisitions were performed with an *m/z* range of 400–1800 with 1 microscan/acquisition. The instrument was operated in data-dependent mode selecting the top five ions in each MS acquisition for further MS/MS acquisition for each MS acquisition. Precursor isolation width was 3 *m/z* units. Singly and doubly charged ions as well as ions with undetermined charge state were excluded from fragmentation. Fragmentation was HCD at an NCE of 25% and selected *m/z* were excluded for 5 seconds until eligible again.

For quantitative analysis the instrument was set to acquire both selected ion monitoring (SIM) and PRM data of the five selected ions as previously described^41^ with some modifications. Briefly, both native peptides and isotope labelled standards were acquired simultaneously by setting the isolation window to 8 *m/z* units. Target values were 1 × 10^6^, maximum isolation time 120 ms, and the resolution setting was 35,000 both for MS and MS/MS acquisitions. No scheduling was employed so the instrument toggled between ten different acquisitions (5 SIM and 5 PRM) throughout the data collection. The cycle time was nevertheless below 2 s ensuring that enough data points were collected for good quantification.

### Qualitative data analysis

The LC-MS/MS.raw files obtained in the data dependent analysis were processed into mascot generic format (.mgf) by the use of mascot distiller software (Matrix science). Settings included: report all ions in their monoprotonated form; minimum signal to noise 5; no summing of spectra; minimum peak width 0.001 u, expected peak width 0.025 u and maximum peak width 0.05 u. Mascot searches were performed with the in-house Mascot server against the 10 isoforms of APP in the Uniprot database (entry P05067). No enzyme restriction was used; Oxidation of Met (+15.9949 u), NeuAcHexHexNAc, (NeuAc)_2_HexHexNAc and (NeuAc)_3_HexHexNAc glycans of Ser/Thr/Tyr (656.2276 u, 947.3230 u and 1238.4184 u) were introduced as allowed modifications. The simultaneous loss of the same glycan masses from the peptide backbone fragment ions, including the arbitrarily Mascot assigned attachment site, was included in the modification parameters. In addition, manual analysis of glycopeptides was conducted by tracing extracted ion chromatograms for selected fragment ions. The general presence of glycopeptides was investigated by tracing oxonium ions such as *m/z* 144.07, specific for HexNAc; and *m/z* 274.09, specific for NeuAc. Also, de-glycosylated peptide ions in common for all glycoforms with the same peptide sequence were assayed. Extracted ion chromatograms were prepared by tracing the first three isotopes of precursor ions in the Xcalibur software.

### Quantitative data analysis of peptide fragment ions

Data processing of the peptide fragment ions was performed with PinPoint v1.3 as previously described^41^ with some modifications. While Aβ1-15 and GalGalNAc-Aβ1-15 were compared to their respective labeled standards, Aβ1-15** and **1**, no standards corresponding to Aβ1-17 and GalGalNAc-Aβ1-17 were available. Therefore these compounds were also compared to Aβ1-15** and **1**. Information on transitions used are given in Supplementary Table 3.

### Quantitative data analysis of oxonium ions

The three MS^2^ spectra with the largest signal intensity of each precursor for endogenous HexHexNAc-Aβ1-15, endogenous HexHexNAc-Aβ1-17 and compound **1** were averaged in the Xcalibur program. The summed absolute intensities of the *m/z* 138.05, 168.07, 186.08 and 204.09 ions arising from endogenous HexHexNAc-Aβ1-15 were divided with the summed intensities of the *m/z* 139.06, 169.07, 187.08 and 205.09 ions from compound **1**. The same procedure was also employed for endogenous HexHexNAc-Aβ1-17 using the internal standard **1**.

## Supplementary information

Supplementary information

Supplementary Table 1

Supplementary Table 2
