# Supplementary material for: Synthetic standard aided quantification and structural characterization of amyloid-beta glycopeptides enriched from cerebrospinal fluid of Alzheimer’s disease patients
Source: Sci Rep. 2019 Apr 2;9:5522. doi: 10.1038/s41598-019-41897-5 (PMC6445081; doi:10.1038/s41598-019-41897-5)
Supplement: Supplementary file 1 — Supplementary information [file 41598_2019_41897_MOESM1_ESM.pdf]

Supplementary Information

Synthetic standard aided quantification and structural characterization of amyloid-beta glycopeptides enriched from cerebrospinal fluid of Alzheimer's disease patients

Jonas Nilsson[1,2], Gunnar Brinkmalm[3], Sherif Ramadan[4,5], Lisa Gilborne[1], Fredrik Noborn[1,2], Kaj Blennow[3,6], Anders Wallin[3], Johan Svensson[7], Mohamed A. Abo-Riya[5], Xuefei Huang[4*], Göran Larson[1,2]*

[1] Laboratory of Clinical Chemistry, Sahlgrenska University Hospital, Gothenburg, Sweden.

[2]Department of Clinical Chemistry and Transfusion Medicine, Institute of Biomedicine, Sahlgrenska Academy, University of Gothenburg, Sweden

[3]Department of Psychiatry and Neurochemistry, Institute of Neuroscience and Physiology, Sahlgrenska Academy, University of Gothenburg.

[4]Departments of Chemistry and Biomedical Engineering, Institute for Quantitative Health, Science and Engineering, Michigan State University, East Lansing, MI, USA.

[5]Chemistry Department, Faculty of Science, Benha University, Benha, Qaliobiya 13518, Egypt.

[6]Clinical Neurochemistry Laboratory, Sahlgrenska University Hospital, Mölndal, Sweden

[7]Department of Internal Medicine, Institute of Medicine Sahlgrenska Academy, University of Gothenburg.

*Corresponding authors: goran.larson@clinchem.gu.se and xuefei@chemistry.msu.edu

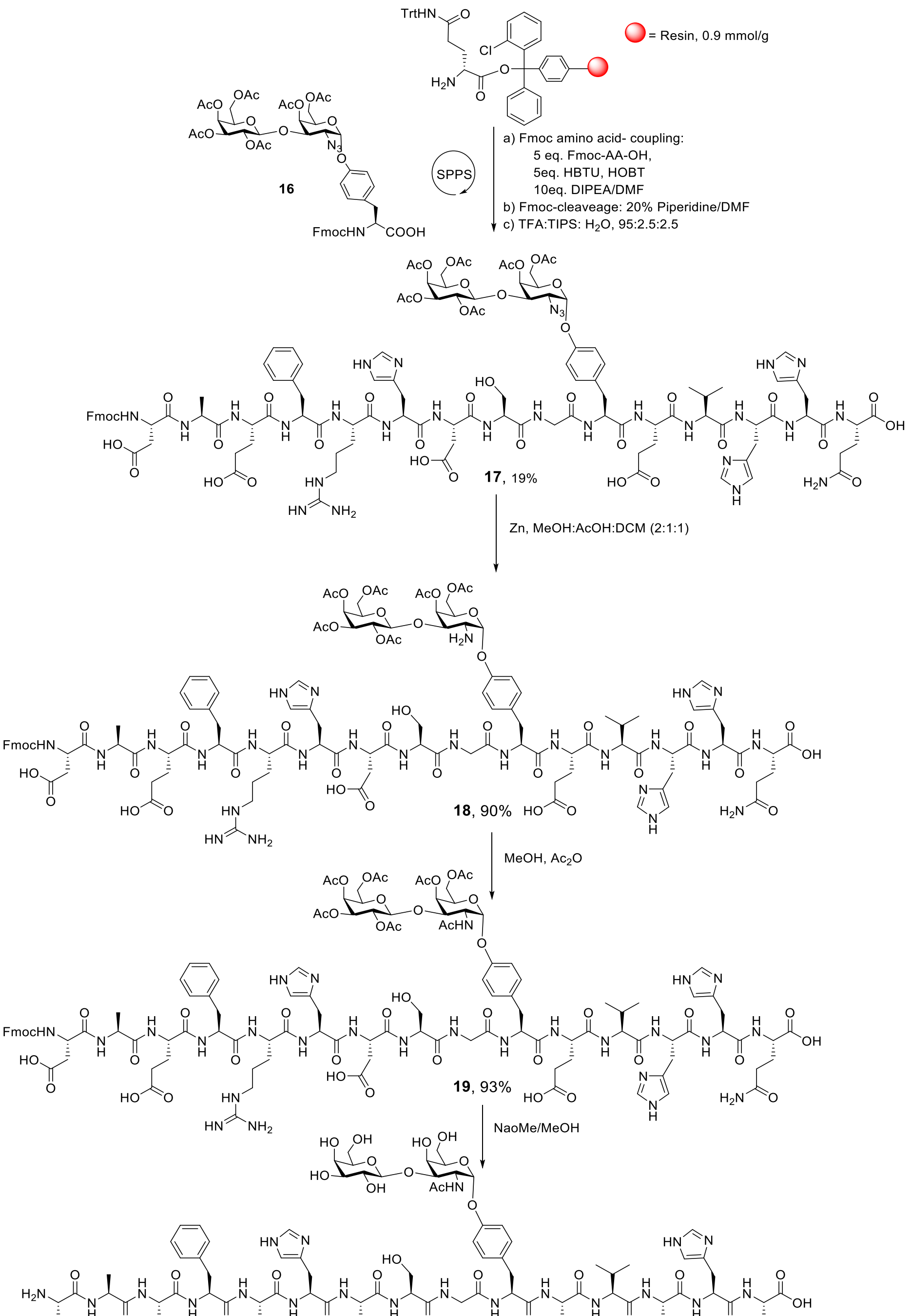

Fig. S1. Solid phase peptide synthesis of Ab1-15 glycopeptide 20.

Commercially available 2-chlorotrityl resin loaded with glutamine was employed for the SPPS. **a**) Fmoc-amino acid (5 eq.) was activated by *O*-(1*H*-benzotriazol-1-yl)-*N*,*N*,*N'*,*N'*-tetramethyluronium hexafluorophosphate (HBTU) (5 eq.), 1-hydroxybenzotriazole (HOBt) (5 eq.) and Hünig's base (*N*,*N*-diisopropylethylamine, DIPEA) (10 eq.). **b**) Fmoc groups were removed with piperidine in DMF (20%). **c**) Cleavage from the solid phase was done with a mixture of trifluoroacetic acid: triisopropyl silane: water (95:2.5:2.5). The azide in **17** was reduced to amine with zinc in methanol:acetic acid:dichloromethane (2:1:1) and acetylated with acetic anhydride in methanol, and **20** was obtained after global deprotection with sodium methoxide in methanol.

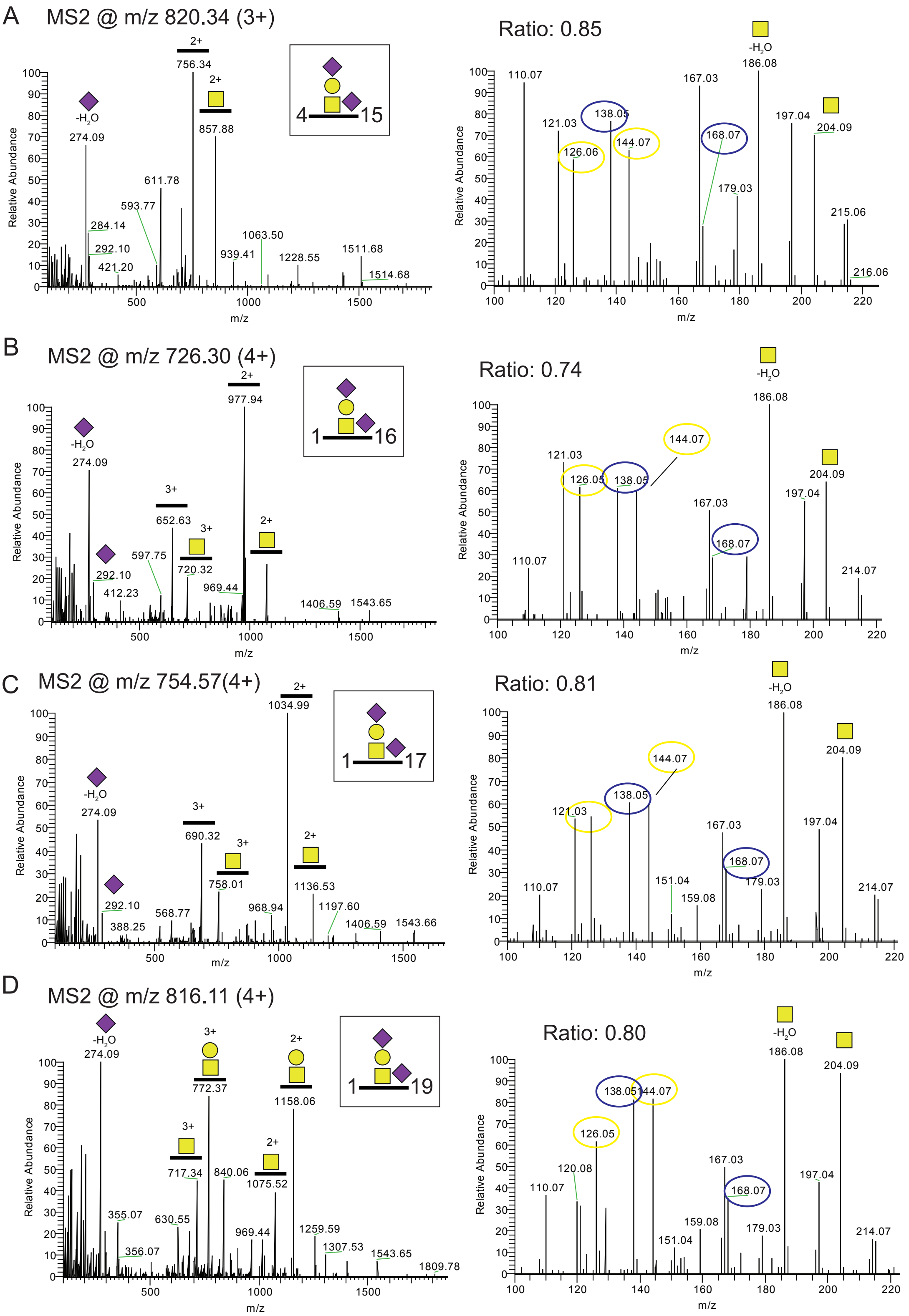

Fig. S2. Endogenous SA$_2$ modified glycopeptides from human CSF. HCD-MS^2 of **A**) SA$_2$-Aβ4-15, **B**) SA$_2$-Aβ1-16, **C**) SA$_2$-Aβ1-17 and **D**) SA$_2$-Aβ1-19. The *m/z* 100-220 region of each MS^2 spectrum are displayed to the right. The (*m/z* 138 + *m/z* 168) / (*m/z* 126 + *m/z* 144) GlcNAc/GalNAc ratios are indicated (yellow and blue circled peaks).

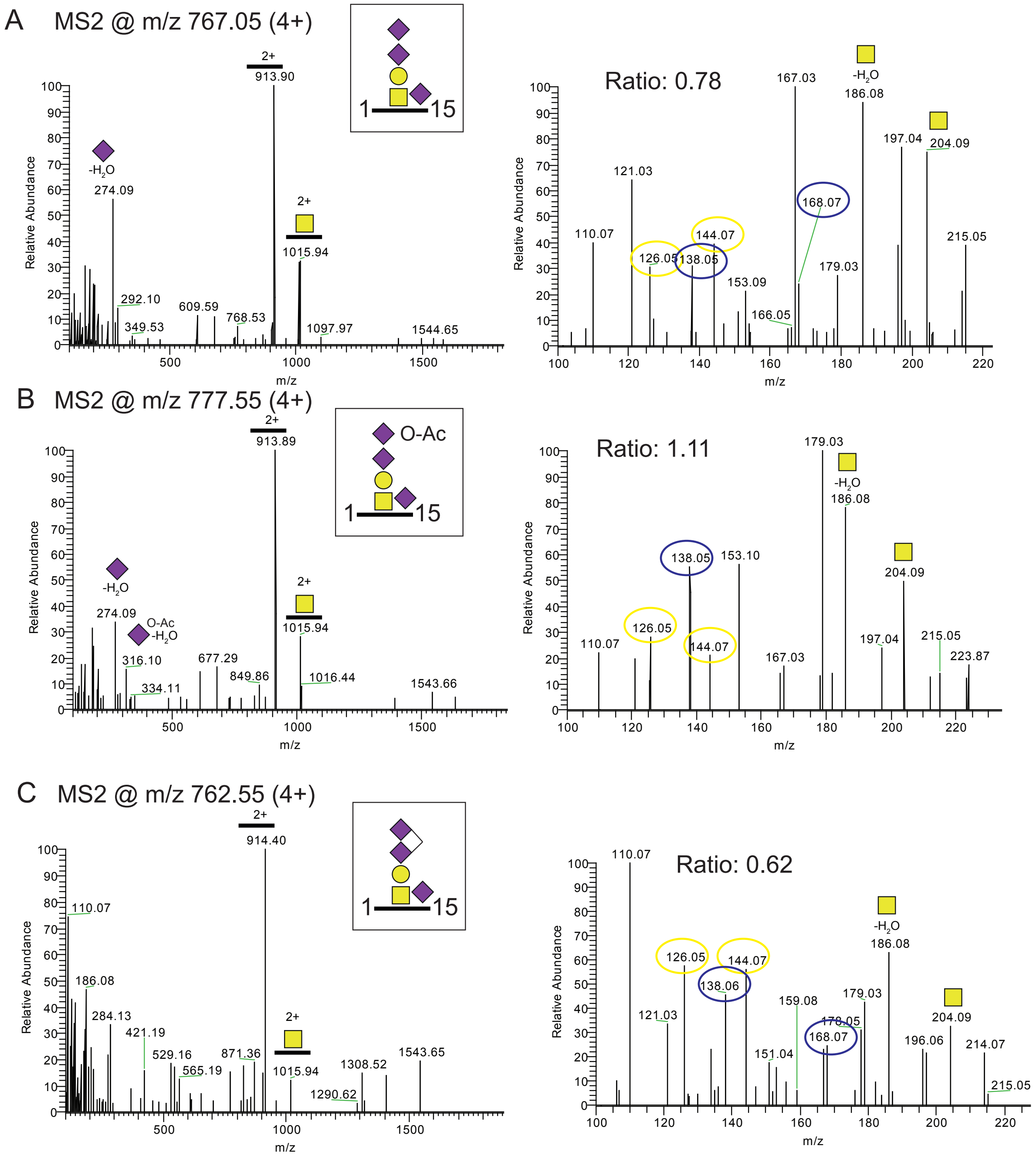

Fig. S3. Endogenous Aβ1-15 glycopeptides from human CSF. HCD-MS2 of **A**) trisialylated SA$_3$-Aβ1-15, **B**) O-acetylated SA$_3$-Aβ1-15 and **C**) dehydrated disialic acid-modified SA$_3$-Aβ1-15 (boxed precursor structures). The *m/z* 100-220 region of each precursor are displayed to the right. The (*m/z* 138 + *m/z* 168) / (*m/z* 126 + *m/z* 144) GlcNAc/GalNAc ratios are indicated (yellow and blue circled peaks).

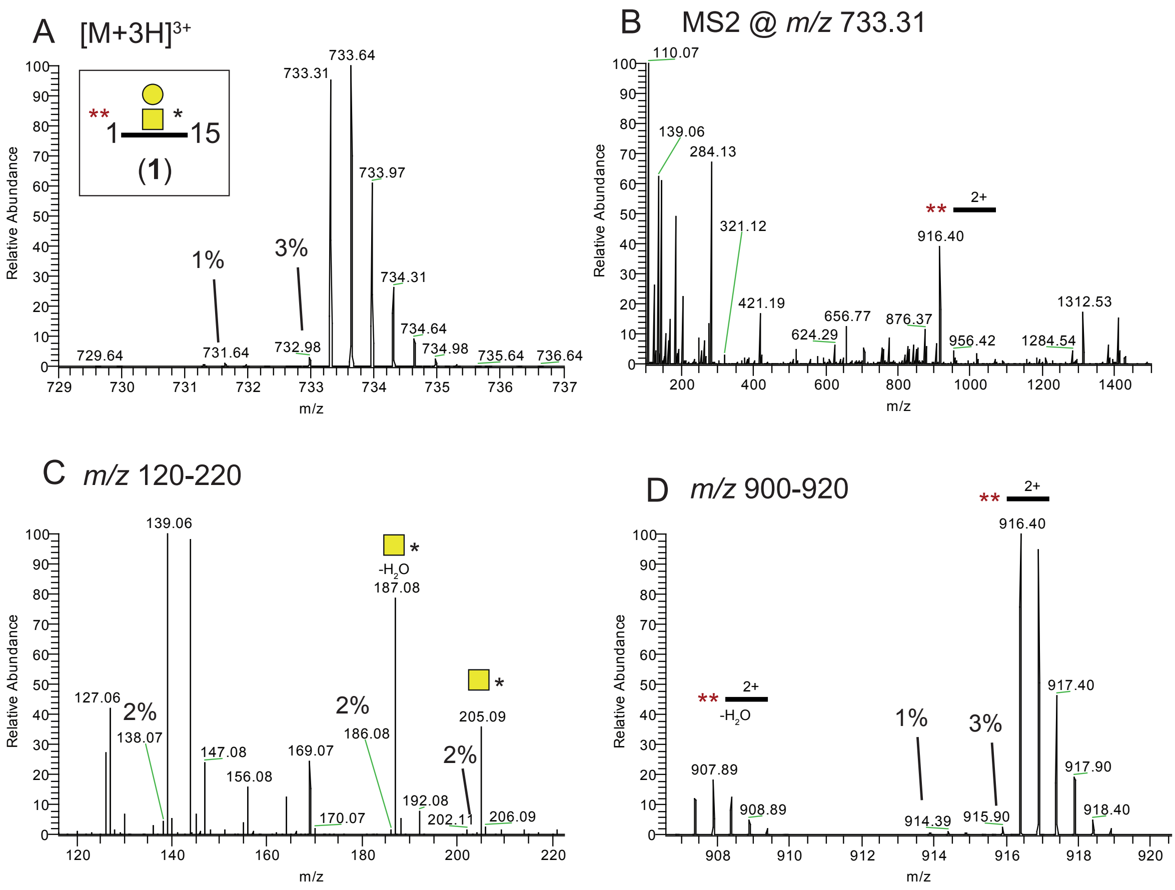

Fig. S4. LC-MS/MS of synthesized Galβ3GalNAc-Aβ1-15 standard 1. (**A**) The precursor MS1 spectrum showed that the relative abundance of the *m/z* 732.98 (-1 u) and *m/z* 731.64 (-5 u) precursors were 3% and 1%, respectively. (**B**) HCD-MS^2 spectrum and (**C**) the *m/z* 120-220 expansion showing that the isotopic purity of the glycan part was 98%. (**D**) Expansion of the *m/z* 900-920 region showing that the isotopic purity of the peptide part was 97% with respect to -1 u, and 99% with respect to the -5 u.

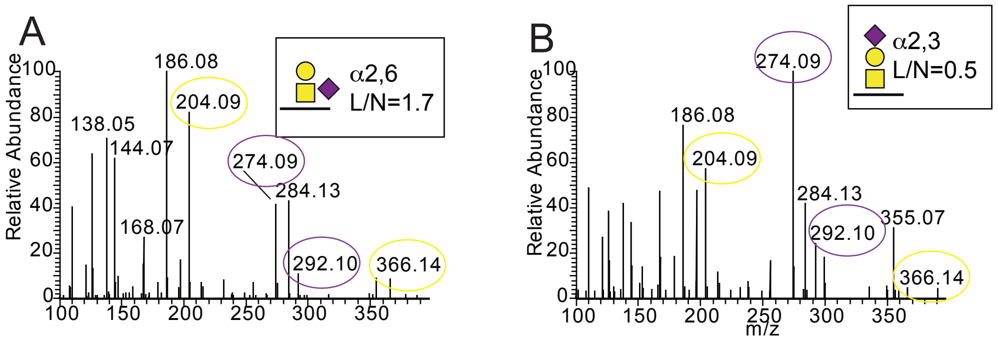

Fig. S5. HCD-MS2 of two SA$_1$-Aβ1-15 glycoforms (**A**) The *m/z* 100-400 region of Galβ3(Neu5Acα2,6)GalNAcα1-*O*- glycoform eluting at 17.96 min of Fig. 4B and (**B**) the Neu5Acα2,3Galβ3GalNAcα1-*O* glycoform eluting at 18.38 min of Fig. 4B. We introduced a LacNAc-to-Neu5Ac oxonium ion ratio (L/N) to distinguish between Neu5Acα2,3 and Neu5Aα2,6 isomers (1). Thereby, the summed intensities of the *m/z* 204 and *m/z* 366 ions (yellow circles) divided by the intensities of the *m/z* 274 and *m/z* 292 ions (purple circles) were shown to be 0.5 (<0.6) for Neu5Acα2,3 and 1.7 (>0.8) for Neu5Acα2,6 containing glycopeptides. The L/N ratios for these MS^2 spectra (boxed) demonstrate the differences in sialic acid linkage glycoforms.

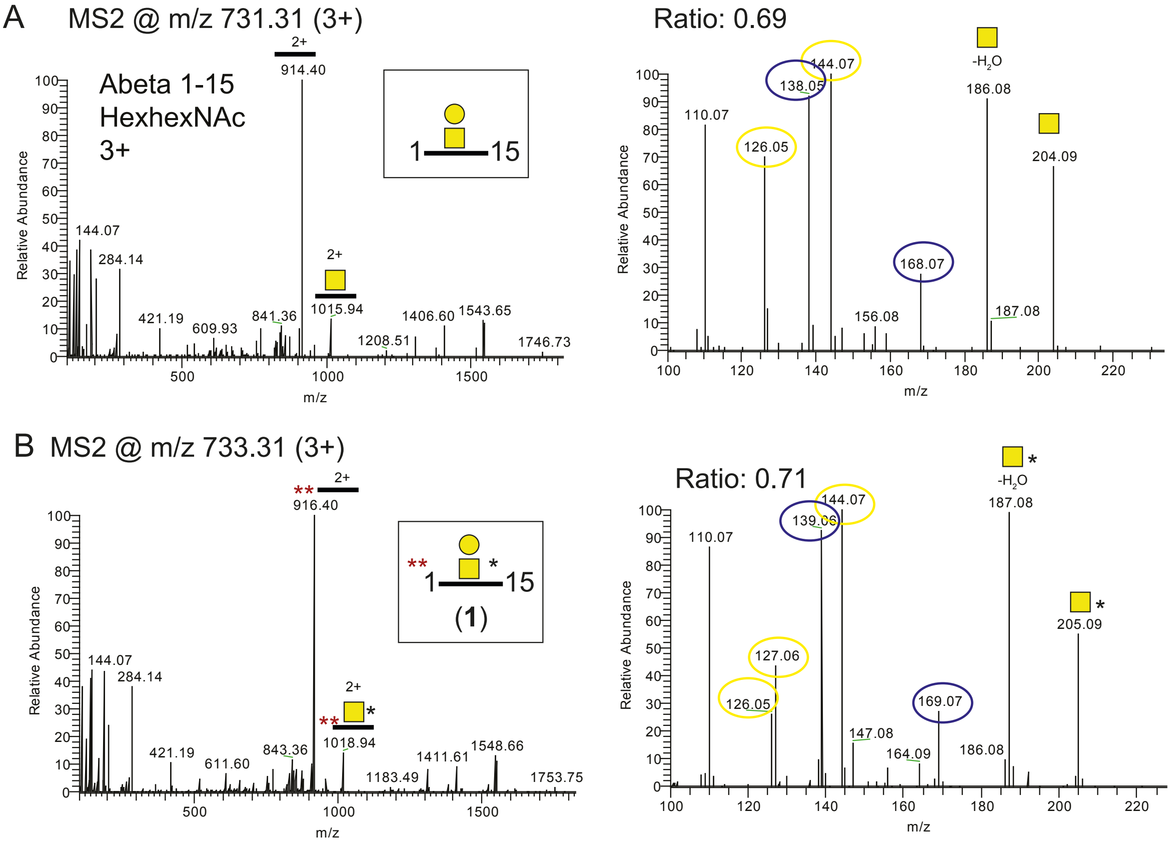

Fig. S6. HCD-MS^2 of co-immunopurified but individually fragmented (A) native Galβ3GalNAc-Aβ1-15 and (B) synthesized Galβ3GalNAc-Aβ1-15 standard 1. The *m/z* 100-220 region of each precursor are displayed to the right. The (*m/z* 138 + *m/z* 168) / (*m/z* 126 + *m/z* 144) GlcNAc/GalNAc ratios are indicated (yellow and blue circled peaks). For **1**, the (*m/z* 139 + *m/z* 169) / (*m/z* 126 + *m/z* 127 + *m/z* 144) ratio was used. This experiment was run according to Route 2 (Fig. 2B) with the exception that data dependent precursor selection was used.

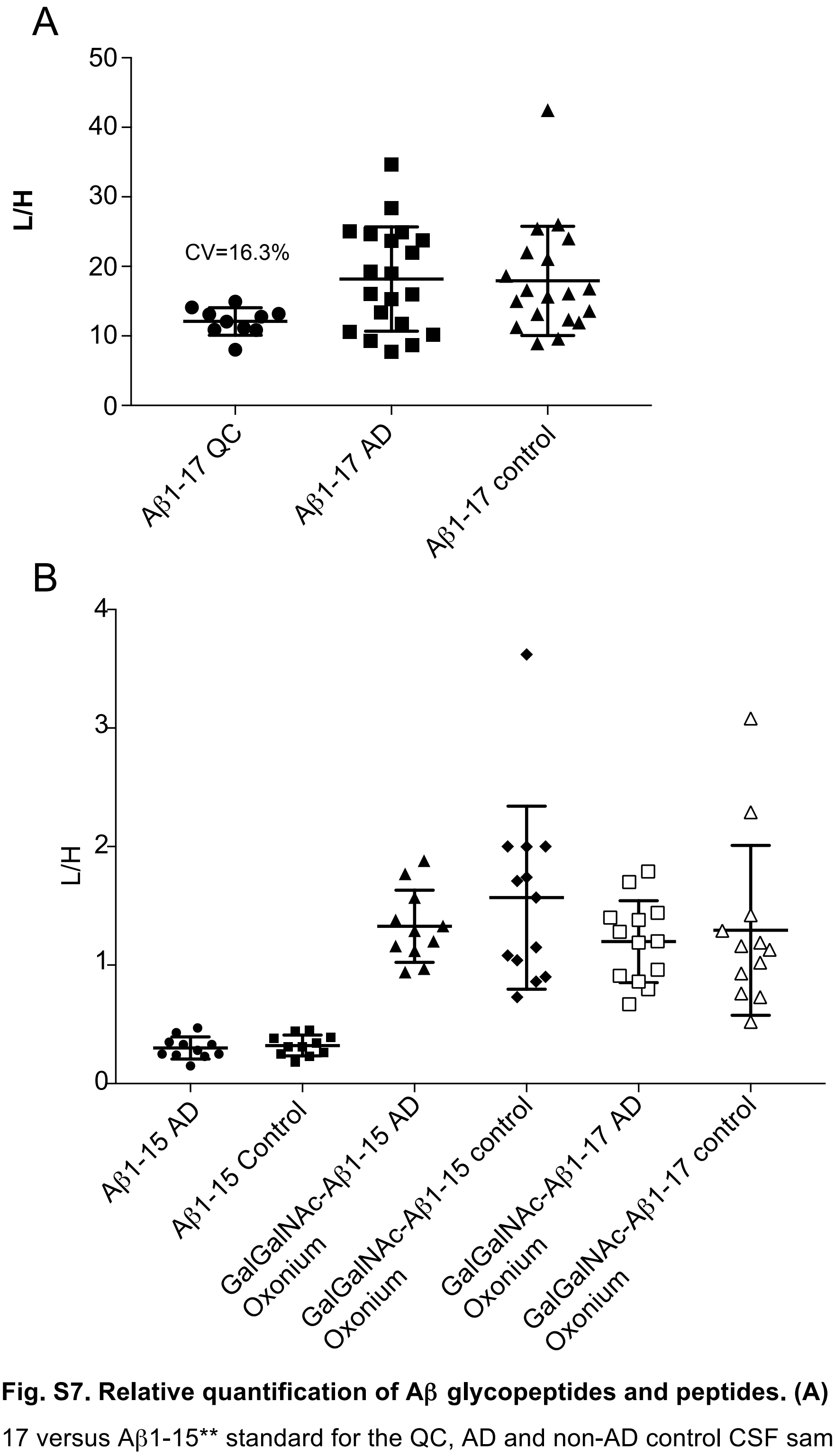

Fig. S7. Relative quantification of Aβ glycopeptides and peptides. (A) Quantification, L/H, of Aβ1-17 versus Aβ1-15** standard for the QC, AD and non-AD control CSF samples. (**B**) Quantification of selected AD and control samples that strictly were classified as AD and control based on their CSF biomarkers P-tau, T-tau and Aβ1-42 values. See Fig. 6B and Supplementary Table 2.

Supplementary Table 3. MS/MS transitions used. For Aβ1-17 and Aβ1-17-HHn, there were no corresponding labeled standards. Therefore the sum of the transition areas for the native peptides were normalized to those of Aβ1-15** and **1**, respectively.

Peptide	Sequence	Percursor *m/z* 3+, native	Percursor *m/z* 3+, labeled	Transitions
Aβ1-15	DAEFRHDSGYEVHHQ	609.600	612.936	1+: b8, b10, b11
Aβ1-15-HHn	DAEFRHDSG<u>Y</u>EVHHQ	731.310	733.314	1+: b6, b10, b11, b12
Aβ1-17	DAEFRHDSGYEVHHQKL	689.993	Ab1-15**<br>612.936	1+: b5, b6, b8, b9, b10, b11, b12<br>2+: b7, b8, b9, b10, b11, b12, b13, b14, b15<br>3+: b16
Aβ1-17-HHn	DAEFRHDSG<u>Y</u>EVHHQKL	811.703	**1**<br>733.314	1+: b6, b10, b11, b12<br>2+: b13, b14. b15

Experimental Procedures

Synthesis of glycan building blocks

1,3,4,6-Tetra-*O*-acetyl-2-deoxy-2-(2, 2, 2-trichloroethoxycarbonylamino)-D-galactopyranoside 4:

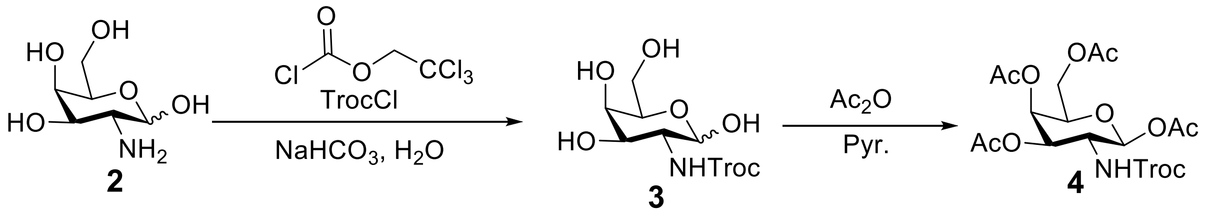

D-Galactosamine hydrochloride **2** (20 g, 92 mmol) was dissolved in water (150 mL), followed by addition of sodium bicarbonate (23.4 g, 278 mmol) and 2,2,2-trichloroethoxycarbonyl chloride (TrocCl) (15.2 mL, 112 mmol) was added dropwise. The reaction mixture was allowed to stir at rt for 3-4 h, then the precipitate was collected via filtration and dried under reduced pressure to afford product **3** as white solid in 94% yield. Then the crude was dissolved in pyridine (90 mL), cooled to 0 °C and followed by slow addition of acetic anhydride (74 mL) and catalytic amount of 4-dimethylaminopyridine (DMAP). The reaction was allowed to stir over night at rt. Upon completion, excess acetic anhydride was quenched by slow addition of MeOH. The mixture was concentrated under vacuum, diluted with ethyl acetate (EtOAc) and washed with 1 M HCl, $NaHCO_3$ and water. The organic phase was then dried over anhydrous Na_2SO_4, filtered and concentrated. After purification by column chromatography (DCM:MeOH = 7:1), compound **4** was obtained as a white solid in 98% yield. Comparison with literature data confirms its identity (2).

***p*-Tolyl 3,4,6-tri-*O*-acetyl-2-(((2,2,2-trichloroethoxy)carbonyl)amino)-2-deoxy-1-thio-*β*-D-galactopyranoside 5:**

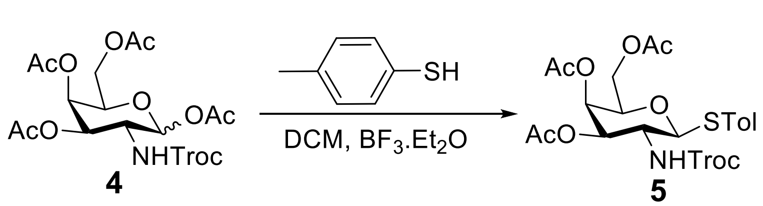

Compound **4** (25.38 g, 48.6 mmol) and 4-methylbenzenethiol (7.84 g) were dissolved in dry DCM (150 mL), followed by slow addition of boron trifluoride etherate (18.45 mL, 145.6 mmol) at 0 °C. The reaction was stirred at r.t. overnight under nitrogen. Upon completion, the reaction was quenched with a sat. $NaHCO_3$, then diluted with DCM (100 mL) and extracted

with DCM (3×100 mL), washed with NaCl, dried over Na_2SO_4, concentrated and purified using column chromatography (Hex:EtOAc, 3:1) to afford the product **5** in 86% yield.

^{1}H NMR ($CDCl_3$, 500MHz): δ 7.39 (d, *J* = 7.3 Hz, 2 H), 7.08 (d, *J* = 7.3 Hz, 2 H), 5.67 (d, *J* = 9.3 Hz, 1 H, H-1), 5.35 (br. s., 1 H), 5.15 (d, *J* = 10.8 Hz, 1 H), 4.85 - 4.73 (m, 2 H), 4.73 - 4.67 (m, 1 H), 4.13 (m, 1 H), 4.10 - 4.05 (m, 1 H), 3.91 (m, 2 H, H-2), 2.30 (s, 3 H, $SPhCH_3$), 2.08 (s, 3 H, $COCH_3$), 2.00 (s, 3 H, $COCH_3$), 1.93 (br. s., 3 H, $COCH_3$). ^{13}C NMR ($CDCl_3$, 125 Hz): δ 170.5, 170.3, 154.2, 138.2, 132.9, 129.6, 128.8, 95.6, 87.4, 74.4, 74.3, 71.1, 66.9, 61.9, 60.5, 51.1, 21.1, 21.1, 20.7, 20.6, 20.6, 14.2. HRMS: $C_{22}H_{26}C_{13}NO_9S$ $[M+H]^+$ calculated: 586.0467, observed: 586.0480.

_p_-Tolyl 2-amino-2-deoxy-1-thio-_β_-D-galactopyranoside 7:

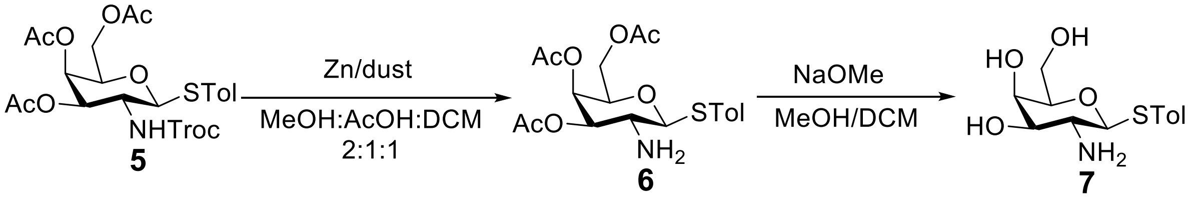

5 6 7

Compound **5** (12.2 g, 20.8 mmol) was dissolved in MeOH/AcOH/DCM (52 mL/26 mL/26 mL), followed by slow addition of zinc dust (14.45 g, 374 mmol). The reaction was allowed to stir for 1h, then filtered over celite, concentrated and purified with column chromatography (Hex:EtOAc, 1:1 to 1:2) to afford product **6** in 80% yield.

^{1}H NMR ($CDCl_3$, 500MHz): δ 7.56 - 7.51 (m, 2 H), 7.15 - 7.08 (m, 2 H), 5.43 - 5.38 (m, 2 H, H-3, H-5), 5.16 (d, J = 9.8 Hz, 1 H, H-1), 4.71 (br. s., 2 H, NH_2), 4.15 - 4.04 (m, 3 H, H5, H-6, H-6'), 3.40 (t, 1 H, H-2), 2.32 (s, 3 H, $SPhCH_3$), 2.03 (s, 3 H, $COCH_3$), 2.02 (s, 3 H, $COCH_3$), 2.00 (s, 3 H, $COCH_3$). ^{13}C NMR ($CDCl_3$, 125 Hz): δ 170.8, 170.5, 170.1, 139.1, 134.2, 129.8, 126.1, 85.3, 74.2, 72.0, 66.5, 61.7, 60.4, 50.5, 21.2, 21.2, 20.7, 20.5, 14.2. HRMS: $C_{19}H_{25}NO_7S$ $[M+NH_4]^+$ calcd: 429.1695, obsd: 429.1711.

Compound **6** (6.9 g) was dissolved in DCM/MeOH (108 mL, 54 mL:54 mL), followed by addition of sodium metal until pH≈14 and the reaction was stirred for 2-3 h at rt. Amberlite resin was added next to adjust the pH to around 7. After filtration, the filtrate was concentrated and dried *in vacuo* to give product **7** in 90% yield, which was used in the next step without further purification.

***p*-Tolyl 2-azido-2-deoxy-1-thio-*β*-D-galactopyranoside 8:**

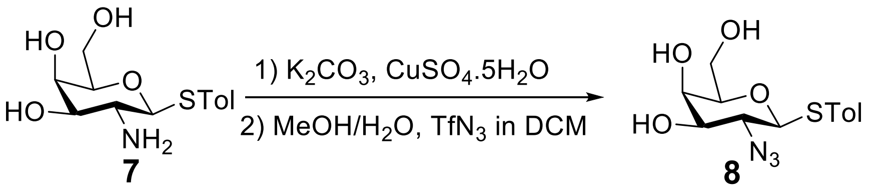

Sodium azide (11.16 g, 171.7 mmol) was dissolved in DCM/H_2O (35 mL/35 mL), followed by addition of trifluoromethanesulfonic anhydride (5.82 mL, 34.2 mmol) at 0 °C. The reaction mixture was stirred vigorously at rt for 2-3 h, then saturated $NaHCO_3$ was added until bubbling ceased. The mixture was extracted with DCM (2×15 mL). Compound **6** (4.82, 16.9 mmol), potassium carbonate (4.75 g, 34.4 mmol), and copper sulfate pentahydrate (0.214 g, 0.857 mmol) were dissolved in MeOH/H_2O (35 mL/35 mL), which was followed by addition of the triflic azide stock solution. Additional MeOH was added until the homogeneous solution was obtained, and the reaction was stirred overnight. Upon completion, the solution was extracted with EtOAc (3×100 mL), dried and concentrated. Silica gel column chromatography was applied (DCM:MeOH, 20:1) to afford the product **8** in 72% yield.

***p*-Tolyl 2-azido-4,6-*O*-benzylidine-2-deoxy-1-thio-*β*-D-galactopyranoside 9:**

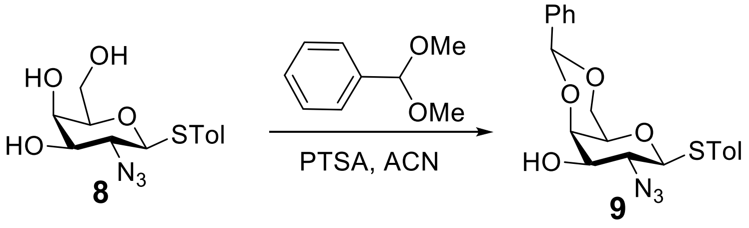

Compound **8** (3.17g, 10.2 mmol) was dissolved in dry acetonitrile (300 mL), and benzaldehyde dimethylacetal (3.1 mL, 20.4 mmol) was added. The pH value was adjusted to 4 with a catalytic amount of *p*-toluenesulfonic acid. The reaction mixture was stirred overnight at room temperature under nitrogen gas. After the reaction completed, it was neutralized with triethylamine (4-5 drops). Then the solvent was concentrated *in vacuo* and diluted with EtOAc, washed with water, dried over Na_2SO_4. Purification of the resulting residue by flash chromatography (Hex:EtOAc:DCM, 10:1:5) gave compound **9** in 90% yield as a colorless amorphous solid.

^{1}H NMR ($CDCl_3$, 500MHz): δ 7.64 (d, J = 8.3 Hz, 2 H), 7.46 - 7.35 (m, 5 H), 7.17 - 7.11 (d, J = 7.8 Hz, 2 H), 5.47 (s, 1 H, Ph-CH), 4.34-4.32 (m, 1 H, H-5), 4.32 - 4.29 (m, 1 H, H-1), 4.03

(d, J = 2.9 Hz, 1 H, H-3), 3.93 (dd, J = 1.5, 12.2 Hz, 1 H, H-6’), 3.57 - 3.46 (m, 2 H, H-2, H-4), 3.36 - 3.30 (m, 1 H, H-6), 2.92 (br. s., 1 H, OH), 2.38 (s, 3 H, $SPhCH_3$). ^{13}C NMR ($CDCl_3$, 125 Hz): δ 138.7, 137.6, 134.6, 129.9, 129.5, 128.3, 126.7, 126.6, 101.3 (PhCH), 85.0 (C1), 74.5, 72.9, 69.7, 69.2, 61.9, 60.5, 53.7, 21.3, 21.1, 14.2. HRMS: $C_{20}H_{21}N_3O_4S$ $[M+NH_4]^+$ calculated: 417.1597, observed: 417.1612.

***p*-Tolyl 2,3,4,6-tetra-*O*-acetyl-1-thio-*β*-D-galactopyranoside 10:**

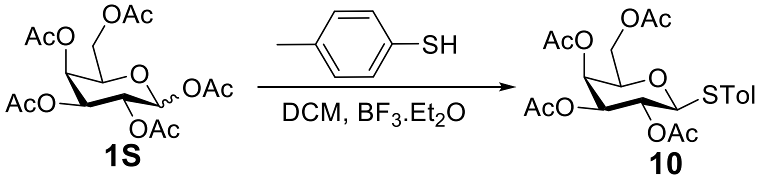

β-D-Galactose pentaacetate **9** (10 g, 25.6 mmol) and 4-methylbenzenethiol (4.14 g, 33.3 mmol) were dissolved in dry DCM, followed by slow addition of boron trifluoride etherate (9.74 mL, 76.9 mmol) at 0 °C. the reaction was stirred at rt overnight. Upon completion, the reaction was quenched with a sat. $NaHCO_3$, then diluted with DCM (100 mL) and extracted with DCM (3×100 mL), washed with NaCl, dried over Na_2SO_4, concentrated and purified using column chromatography (Hex:EtOAc, 3:1) to afford the product **10** in 90% yield.

^1H NMR ($CDCl_3$, 500 MHz): δ 7.44 - 7.39 (m, 2 H), 7.15 - 7.10 (m, 2 H), 5.41 (dd, J = 1.0, 3.4 Hz, 1 H, H-4), 5.22 (t, J = 10.0 Hz, 1 H, H-2), 5.04 (dd, J = 3.4, 9.8 Hz, 1 H, H-3), 4.65 (d, J = 10.3 Hz, 1 H, H-1), 4.19 (dd, J = 6.8, 11.2 Hz, 1 H, H-6’), 4.11 (dd, J = 6.4, 11.2 Hz, 1 H, H-6), 3.94 - 3.88 (m, 1 H, H-5), 2.35 (s, 3 H, $SPhCH_3$), 2.12 (s, 3 H, $COCH_3$), 2.10 (s, 3 H, $COCH_3$), 2.05 (s, 3 H, $COCH_3$), 1.97 (s, 3 H, $COCH_3$). ^{13}C NMR ($CDCl_3$, 125 Hz): δ 170.4, 170.2, 170.1, 169.4, 138.5, 133.1, 129.6, 128.6, 86.9 (C1), 74.3, 72.0, 67.3, 67.2, 61.6, 21.2, 20.9, 20.7, 20.6, 20.6.

***p*-Tolyl 2-azido-4,6-*O*-benzylidene-2-deoxy-3-*O*-[2,3,4,6-tetra-*O*-acetyl-*β*-D-galactopyranosyl]-1-thio-*β*-D-galactopyranoside 11:**

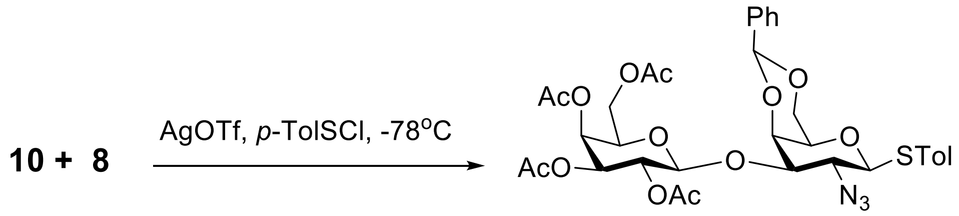

11

p-Tolyl 2,3,4,6-tetra-*O*-acetyl-1-thio-*β*-D-galactopyranoside (donor) **10** (0.64 g, 1.4 mmol) and *p*-tolyl 2-azido-4,6-*O*-benzylidine-2-deoxy-1-thio-*β*-D-galactopyranoside (acceptor) **8** (979 mg, 1.81 mmol) were each separately co-evaporated with toluene (3×30 mL) and dried under high vacuum. The glycosyl donor **10** was dissolved in dry DCM (15 mL), and activated molecular sieves (0.8 g, 4 Å) were added. The suspension was stirred for 1 h at ambient temperature under nitrogen, then cooled to -78 °C. A solution of AgOTf (1.085 g, 4.2 mmol) in anhydrous Et_2O/DCM (8 mL/1 mL) was added to reaction solution without touching the wall of the flask. After 5 min, orange colored *p*-TolSCl (0.203 mL, 1.38 mmol) was directly added by a microsyringe to the reaction mixture to avoid freezing on flask wall. This color disappeared in few seconds indicating consumption of *p*-TolSCl promoter. After 5 min, TLC indicated the complete activation of donor. Then a solution of the acceptor and 2,4,6-tri-*t*-butyl pyrimidine (TTBP, 1 eq.) in anhydrous DCM were added to the reaction via syringe. The reaction was allowed to stir for 1 h at -78 °C then the temperature was raised to -10 °C with continued stirring for another 1 h. Finally, the reaction was quenched by Et_3N (3-4 drops), diluted with DCM and filtered over celite. The DCM solution was washed with aqueous solutions of $NaHCO_3$ and NaCl respectively, dried over Na_2SO_4, concentrated and purified by silica gel flash chromatography (Hex:EtOAc, 3:1) to afford the product **11** as a white solid in 70% yield.

^{1}H NMR ($CDCl_3$, 500 MHz): δ 7.60 - 7.56 (m, 2 H), 7.45 - 7.36 (m, 5 H), 7.04 - 7.00 (m, 2 H), 5.72 (d, J = 4.9 Hz, 1 H, H-1b), 5.52 (s, 1 H, PhCH), 5.39 (dd, J = 2.4, 3.4 Hz, 1 H, H-3b), 5.00 (dd, J = 3.4, 6.8 Hz, 1 H), 4.40 - 4.35 (m, 2 H, H-1a), 4.29 - 4.24 (m, 3 H, H-2b,H-4b), 4.12 - 4.06 (m, 2 H), 4.03 (d, J = 10.8 Hz, 1 H), 3.67 (d, J = 3.4 Hz, 1 H), 3.64 (d, J = 9.8 Hz, 1 H), 3.48 - 3.44 (m, 1 H), 2.32 (s, 3 H, $SPhCH_3$), 2.06 - 2.05 (m, 3 H, $COCH_3$), 2.04 (s, 3 H, $COCH_3$), 2.03 (s, 3 H, $COCH_3$), 1.75 (s, 3 H, $COCH_3$). ^{13}C NMR ($CDCl_3$, 125 Hz): δ 171.2, 170.5, 170.0, 169.8, 138.7, 137.7, 134.8, 129.8, 129.8, 129.1, 128.2, 128.1, 126.6, 126.5, 126.0, 120.8, 101.4, 101.0 (PhC), 97.7 (C-1b), 90.6, 85.2 (C-1a), 74.8, 74.3, 73.1, 71.0, 69.8,

69.7, 69.3, 69.2, 69.1, 68.3, 67.2, 65.6, 61.8, 61.2, 60.4, 59.1, 22.8, 21.2, 21.1, 20.8, 20.7, 20.7, 20.7, 20.5, 14.2. HRMS: $C_{34}H_{39}N_3O_{13}S$ $[M+NH_4]^+$ calculated: 747.2547, observed: 747.2602.

***p*-Tolyl 4,6-di-*O*-acetyl-2-azido-2-deoxy-3-*O*-[2,3,4,6-tetra-*O*-acetyl-*β*-D-galactopyranosyl]-1-thio-*β*-D-galactopyranoside 13:**

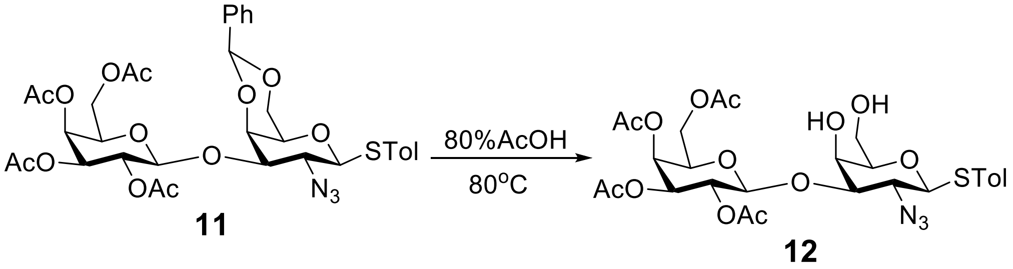

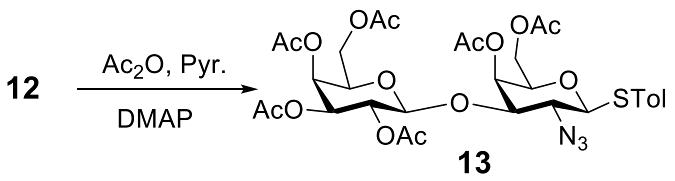

A solution of disaccharide **11** (700 mg) in aqueous acetic acid (80%, 30 mL) was stirred at 70 °C for 1 h. Toluene (20 mL) was added, and the reaction mixture was concentrated *in vacuo* and subsequently co-evaporated with toluene (3×15 mL). The resulting crude product was purified by silica gel flash chromatography (EtOAc:Hex, 2:1) to give *p*-tolyl 2-azido-2-deoxy-3-*O*-[2,3,4,6-tetra-*O*-acetyl-6-*β*-D-galactopyranosyl]-1-thio-*β*-D-galactopyranoside **12** as a colorless amorphous solid in 90% yield.

^{1}H NMR ($CDCl_3$, 500 MHz): δ 7.45 - 7.42 (m, 2 H), 7.11 - 7.08 (m, 2 H), 5.34 (d, J = 3.4 Hz, 1 H), 5.20 (dd, J = 8.1, 10.5 Hz, 1 H), 4.99 (dd, J = 3.4, 10.8 Hz, 1 H), 4.66 (d, J = 8.3 Hz, 1 H, H-1b), 4.33 (d, J = 10.3 Hz, 1 H, H-1a), 4.07 (d, J = 6.8 Hz, 1 H), 4.06 - 4.04 (m, 1 H), 4.02 - 4.00 (m, 1 H), 3.93 - 3.89 (m, 2 H), 3.75 (dd, J = 4.9, 11.7 Hz, 1 H), 3.55 (t, J = 10.0 Hz, 1 H), 3.49 - 3.42 (m, 2 H), 2.83 (br. s., 1 H), 2.78 (br. s., 1 H), 2.30 (s, 3 H, $SPhCH_3$), 2.11 (s, 3 H, $COCH_3$), 2.03 (s, 3 H, $COCH_3$), 2.00 (s, 3 H, $COCH_3$), 1.94 (s, 3 H, $COCH_3$). ^{13}C NMR ($CDCl_3$, 125 Hz): δ 171.2, 170.5, 170.1, 170.1, 169.5, 138.6, 133.4, 129.9, 127.3, 101.8 (C-1b), 86.7 (C-1a), 83.3, 78.0, 71.1, 70.6, 68.4, 67.7, 67.0, 62.2, 61.5, 60.8, 60.4, 21.1, 21.0, 20.6, 20.5, 14.1. HRMS: $C_{27}H_{35}N_3O_{13}S$ $[M+H]^+$ calculated: 642.1963, observed: 642.1975.

Compound **12** was dissolved in pyridine and cooled to 0 °C followed by slow addition of acetic anhydride and catalytic amount of DMAP was added. The solution was stirred at ambient temperature for 5 h, quenched with MeOH (15 mL). Toluene (15 mL) was added and the solution was concentrated *in vacuo*. Purification of the residue by column chromatography on silica gel (Hex:EtOAc, 3:1 to 1:1) afforded the protected compound **13** as a colorless, amorphous solid (92%).

^{1}H NMR ($CDCl_3$, 500 MHz): δ 7.47 - 7.43 (m, 2 H), 7.13 - 7.08 (m, 2 H), 5.32 (d, *J* = 2.9 Hz, 2 H), 5.12 (dd, *J* = 7.8, 10.8 Hz, 1 H), 4.96 (dd, *J* = 3.4, 10.8 Hz, 1 H), 4.66 (d, *J* = 7.8 Hz, 1 H, H-1b), 4.35 (d, *J* = 9.3 Hz, 1 H, H-1a), 4.11 - 3.98 (m, 4 H), 3.86 (s, 1 H), 3.75 - 3.69 (m, 1 H), 3.56 - 3.47 (m, 2 H), 2.32 (s, 3 H), 2.10 (s, 3 H), 2.03 (d, *J* = 2.4 Hz, 6 H), 2.01 (s, 6 H), 1.94 (s, 3 H). ^{13}C NMR ($CDCl_3$, 125 Hz): δ 165.8, 165.6, 165.5, 165.3, 164.9, 164.6, 134.1, 129.1, 124.9, 122.4, 96.6, 81.9, 74.8, 70.4, 66.1, 65.9, 64.0, 63.3, 62.0, 57.9, 56.6, 56.2, 55.6, 16.4, 16.0, 16.0, 15.9, 15.9, 15.9, 15.9, 15.8, 15.8, 15.8. HRMS: $C_{31}H_{39}N_3O_{15}S$ $[M+NH_4]^+$ calculated: 743.2446, observed: 743.2530.

N$^{\alpha}$-[(9H-fluoren-9-ylmethoxy)-carbonyl]-L-tyrosine *tert*-butyl ester 14:

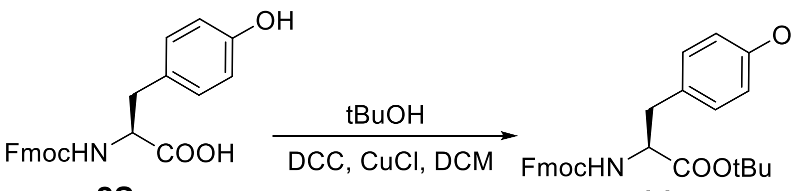

2S **14**

Dicyclohexylcarbodiimide (9.3 g, 45 mmol), *tert*-butanol (4.4 g, 58 mmol) and copper-(I)-chloride (0.1 g, 1 mmol) were mixed together and stirred for 5d. This deep green suspension solution diluted with anhydrous DCM and the *N*-protected amino acid **2S** (14 mmol) in DCM (30 mL) was added drop wise. The mixture was stirred for 4 h. Upon completion, the reaction mixture was filtered to remove the precipitated urea and the organic layer was washed with sat. $NaHCO_3$ three times, dried with Na_2SO_4 and concentrated *in vacuo*. The product was purified with flash column chromatography (Hex: EtOAc) to yield pure hydroxyl amino acid **14** as a white powder in 92% yield.

^{1}H NMR ($CDCl_3$, 500 MHz): δ 7.79 (d, J = 7.3 Hz, 2 H), 7.63 - 7.57 (m, 2 H), 7.42 (m, 2 H), 7.23 - 7.17 (m, 2 H), 7.05 - 7.00 (m, 2 H), 6.79 - 6.73 (m, 2 H), 5.44 (d, J = 8.3 Hz, 1 H, NH), 4.57 (d, J = 7.8 Hz, 1 H, Tyr-CH), 4.45 (d, J = 7.3 Hz, 1 H, Fmoc-CH_2), 4.39 (d, J = 6.8 Hz, 1 H, Fmoc-CH_2), 4.26 - 4.21 (m, 1 H, Fmoc-CH), 3.05 (dd, J = 6.1, 8.6 Hz, 2 H, Tyr-CH_2), 1.47 (s, 9 H, tBu). ^{13}C NMR ($CDCl_3$, 125 Hz): δ 171.0, 155.9, 155.2, 143.8, 143.7, 141.3, 130.6,

129.1, 128.3, 127.8, 127.5, 127.1, 125.3, 125.2, 125.1, 120.0, 120.0, 115.4, 82.7, 67.1, 55.4, 47.1, 37.6, 28.0, 21.6. HRMS: $C_{28}H_{29}NO_5$ $[M+NH_4]^+$ calculated: 460.2118, observed: 460.2222.

***N*^α^-(9*H*-Fluoren-9-yl)-methoxycarbonyl-*O*-(4,6-di-*O*-acetyl-2-azido-2-deoxy-3-*O*-[2,3,4,6-tetra-*O*-acetyl-*β*-D-galactopyranosyl]-*α*-D-galactopyranosyl)-L-tyrosine *tert*-butyl ester 15:**

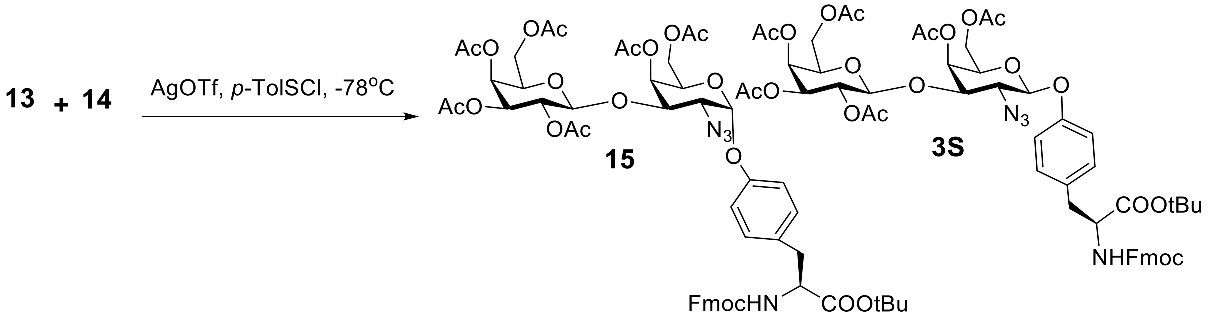

A mixture of disaccharide donor **13** (0.319 g, 0.44 mmol) and amino acid derivative acceptor **14** (0.209 g, 0.53 mmol) were dissolved and co-evaporated with anhydrous toluene (2×20 mL) and dried in high vacuum. Then the mixture was dissolved in anhydrous DCM (15 mL). Molecular sieves (1 g, 3 Å) and TTBP (0.11 g, 0.44 mmol) were added and the suspension was stirred for 1 h at r.t. under nitrogen. The mixture was cooled to -78 °C and treated with silver triflate (0.395 mg, 1.54 mmol) in anhydrous Et_2O/DCM (4 mL/0.5 mL). After 5 min, orange colored *p*-TolSCl (0.203 mL, 1.38 mmol) was directly added to the reaction mixture by a microsyringe to avoid frozen on flask wall. This color disappeared in few seconds indicating consumption of *p*-TolSCl promoter. After 5 min, TLC indicated the complete activation of donor. Then a solution of the acceptor in anhydrous DCM was added to the reaction via syringe. The reaction was allowed to stir for 1 h at -78 °C then the temperature raised to -10 °C and stirring was continued for another 1 h. Finally, the reaction was quenched by DIPEA (3-4 drops), diluted with DCM and filtered over celite. The DCM solution was washed with water, dried over Na_2SO_4, concentrated and purified by silica gel flash chromatography (Hex:EtOAc, 3:1) to afford the product **15** as a white solid in 75% yield and the *β* isomer **3s** in 12% yield.

For *α*-isomer **15**: ^1H NMR ($CDCl_3$, 500 MHz): δ 7.81 - 7.76 (m, 2 H), 7.61 - 7.54 (m, 2 H), 7.44 - 7.39 (m, 2 H), 7.35 - 7.30 (m, 2 H), 7.09 (d, J = 8.3 Hz, 2 H), 7.04 - 7.00 (m, 2 H), 5.53 (d, J = 2.9 Hz, 2 H, H-1a,), 5.40 (d, J = 3.4 Hz, 1 H), 5.27 - 5.21 (m, 2 H), 5.05 (dd, J = 3.4, 10.3 Hz, 1 H), 4.78 (d, J = 7.8 Hz, 1 H, H-1b), 4.53 (d, J = 7.8 Hz, 1 H), 4.46 (dd, J = 6.8,

10.8 Hz, 1 H), 4.36 - 4.28 (m, 2 H), 4.20 (m, 3 H), 4.15 - 4.09 (m, 2 H), 4.00 - 3.94 (m, 2 H), 3.78 (dd, J = 3.4, 10.3 Hz, 1 H), 3.13 - 2.99 (m, 2 H, Tyr-CH_2), 2.18 (s, 3 H, $COCH_3$), 2.16 (s, 3 H, $COCH_3$), 2.11 (s, 3 H, $COCH_3$), 2.07 (s, 3 H, $COCH_3$), 2.00 (s, 3 H, $COCH_3$), 1.92 (s, 3 H, $COCH_3$), 1.45 (s, 9 H, tBu). ^{13}C NMR ($CDCl_3$, 125 Hz): δ 170.5, 170.4, 170.4, 170.3, 170.1, 169.7, 169.5, 155.5, 155.3, 143.8, 143.8, 141.3, 141.3, 137.9, 133.9, 131.0, 130.8, 129.7, 129.0, 128.2, 127.8, 127.7, 127.1, 127.1, 125.3, 125.2, 125.0, 120.0, 120.0, 116.9, 101.6 (C-1b,($^1J_{C\text{-}H}$=160 Hz)), 97.3 (C-1a, ($^1J_{C\text{-}H}$=180 Hz)), 82.5, 74.8, 70.9, 70.8, 69.2, 68.8, 68.2, 66.8, 66.8, 62.4, 61.1, 59.3, 55.1, 47.2, 37.5, 28.0, 21.5, 20.8, 20.7, 20.7, 20.6, 20.6. HRMS: $C_{52}H_{60}N_4O_{20}$ $[M+NH_4]^+$ calculated: 1078.4145, observed: 1078.4321.

For *β*-isomer **3S:** ^1H NMR (500 MHz, $CDCl_3$) δ 7.78 (d, *J* = 7.6 Hz, 2H), 7.57 (d, *J* = 8.1 Hz, 2H), 7.42 (m, 2H), 7.34 – 7.31 (m, 2H), 7.10 (d, *J* = 8.4 Hz, 2H), 7.01 – 6.98 (m, 2H), 5.38 (m, 2H), 5.28 (d, *J* = 8.1 Hz, 1H), 5.19 (m, 2H), 5.03 (m, 2H), 4.73 (m, 2H, H-1b, H-1a), 4.56 – 4.51 (m, 1H), 4.45 (dd, *J* = 10.6, 7.1 Hz, 1H), 4.31 (dd, *J* = 10.6, 7.3 Hz, 1H), 4.22 – 4.15 (m, 4H), 4.13 – 4.04 (m, 4H), 3.92 – 3.86 (m, 3H), 3.83 – 3.80 (m, 1H), 3.57 (dd, *J* = 10.4, 3.5 Hz, 1H), 3.10-3.06 (m, 2H), 2.18 (s, 3H, $COCH_3$), 2.16 (s, 3H, $COCH_3$), 2.11 (s, 3H, $COCH_3$), 2.07 (s, 3H, $COCH_3$), 2.05 (s, 3H, $COCH_3$), 2.00 (s, 3H, $COCH_3$), 1.45 (s, 9H, tBu). ^{13}C NMR ($CDCl_3$, 125 Hz): δ 170.43, 169.79, 169.37, 130.70, 129.03, 128.22, 127.06, 125.29, 120.01, 117.04, 101.55 (C-1b, ($^1J_{C\text{-}H}$=160 Hz)), 100.82 (C-1a, ($^1J_{C\text{-}H}$=162.5 Hz)), 82.54, 71.74, 70.84, 70.59, 68.75, 66.92, 66.75, 62.91, 62.29, 61.02, 28.02, 21.47, 20.73, 20.70, 20.65, 20.60.

N*^α^-(9*H*-Fluoren-9-yl)-methoxycarbonyl-*O*-(2-acetamido-4,6-di-*O*-acetyl-2-deoxy-3-*O*-[2,3,4,6-tetra-*O*-acetyl-*β*-D-galactopyranosyl]-*α*-D-galactopyranosyl)-L-tyrosine 16*:

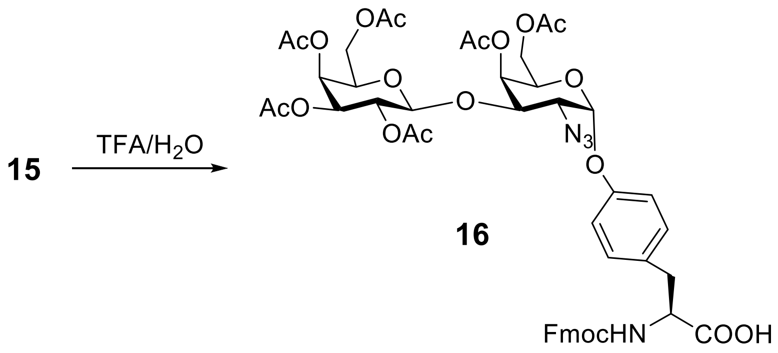

A solution of Fmoc-Tyr(*β*-Ac_4Gal-(1-3)-*α*-Ac_2GalNAc)-O^{t}Bu **15** (0.51 g, 0.5 mmol) in trifluoroacetic acid/H_2O (20 mL/2 mL) was stirred for 3 h at rt. Then the solvents were removed *in vacuo*, and the residue was co-evaporated with toluene (5×20 mL). The crude

product was purified by flash chromatography on silica gel (EtOAc–Hex, 4:1) to give **16** as a colorless, amorphous solid in 98% yield.

^{1}H NMR ($CDCl_3$, 500 MHz): δ 7.76 (d, J = 7.3 Hz, 2 H), 7.52 (d, J = 7.8 Hz, 1 H), 7.54 (d, J = 7.8 Hz, 1 H), 7.39 (t, J = 7.3 Hz, 2 H), 7.29 (t, J = 7.3 Hz, 2 H), 7.08 (d, J = 8.8 Hz, 2 H), 7.00 (d, J = 8.8 Hz, 2 H), 5.50 (dd, J = 3.2, 7.6 Hz, 2 H, H-1a), 5.38 (d, J = 3.4 Hz, 1 H, H-5b), 5.29 (t, J = 3.9 Hz, 1 H, H-4b), 5.21 (dd, J = 7.8, 10.3 Hz, 1 H, H-2b), 5.04 (dd, J = 3.2, 10.5 Hz, 1 H, H-6b), 4.76 (d, J = 7.8 Hz, 1 H, H-1b), 4.66 (d, J = 7.3 Hz, 1 H, H-3b), 4.49 - 4.41 (m, 1 H), 4.33 (dd, J = 7.1, 10.5 Hz, 1 H), 4.27 (dd, J = 3.2, 10.5 Hz, 1 H, H-2a), 4.21 - 4.13 (m, 3 H, H-3a), 4.12 - 4.05 (m, 2 H, H-4a), 3.98 - 3.91 (m, 2 H), 3.76 (dd, J = 3.4, 10.5 Hz, 1 H), 3.22 - 3.11 (m, 1 H, Tyr-CH_2), 3.11 - 3.01 (m, 1 H, Tyr-CH_2), 2.16 (s, 3 H, $COCH_3$), 2.14 (s, 3 H, $COCH_3$), 2.09 (s, 3 H, $COCH_3$), 2.05 (s, 3 H, $COCH_3$), 1.99 (s, 3 H, $COCH_3$), 1.88 (s, 3 H, $COCH_3$). ^{13}C NMR ($CDCl_3$, 125 Hz): δ 170.7, 170.6, 170.4, 170.2, 169.9, 169.7, 155.8, 155.4, 143.6, 141.3, 141.3, 130.6, 130.4, 127.8, 127.1, 125.1, 125.0, 120.1, 120.0, 117.1, 101.6 (C-1b,($^1J_{C-H}$=160 Hz)), 97.1 (C-1a, ($^1J_{C-H}$=180 Hz)), 74.8, 70.8, 69.2, 68.8, 68.2, 67.0, 66.8, 62.4, 61.1, 59.2, 54.6, 47.1, 20.8, 20.7, 20.7, 20.6. HRMS: $C_{48}H_{52}N_4O_{20}$ $[M+Na]^+$ calculated: 1027.3073, observed: 1027.3124.

General method for the peptide synthesis:

All glycopeptide syntheses were synthesized according to the Fmoc-chemistry based solid phase peptide synthesis procedure. Resin with pre-loaded amino acid was loaded into a plastic syringe fitted with a filter and swelled in DCM for 1h. For the coupling reactions, the Fmoc-amino acids (5 eq.) was activated by *O*-(benzotriazol-1-yl)-N, N, N’, N’-tetramethyluronium hexafluorophosphate (HBTU, 5 eq), 1-hydroxybenzotriazole (HOBt, 5 eq), di-isopropylethylamine (DIPEA, 10 eq) and anhydrous dimethylformamide (DMF, 5 mL) for 30 min. Then this mixture containing activated Fmoc amino acid was transferred to the syringe containing the resin preloaded with amino acid and allowed to rotate on a rotator for 30 min. at 70 °C. After completion of coupling, the resin was washed with dichloromethane (DCM, 3×5 mL) and DMF (3×5 mL) for 1 min. each time, followed by cleavage of the Fmoc group by treatment of the resin with a solution of piperidine (20%) in DMF for at least 2×20 min at rt. After every coupling step, unreacted amino groups were capped by treatment with a mixture of Ac_2O (0.5 mL), and DIPEA (1 mL) in DMF (3.5 mL) (capping reagent) for 2×15 min. each. For addition of the glycosyl amino acid, the glycosylated tyrosine building blocks

(2 eq.) were dissolved in DMF and activated with HATU (2 eq.), HOAt (2 eq.) and DIPEA (4 eq.) and the syringe was agitated on the rotator for 2h at 70 °C. After completion of the peptide chain, the resin was washed and the glycopeptide was cleaved from the resin by treatment with trifluoroacetic acid (TFA)/triisopropyl silane (TIPS)/H_2O (95%: 2.5%: 2.5%) solution for 2.5 h. After filtration, the resins were washed with TFA (2×10 mL), and the volume of the combined filtrates was concentrated to 1 mL, then absolute Et_2O (20 mL) at 0 °C was added dropwise to the mixture. The precipitates were separated from the mother liquor by centrifugation and washed with cold Et_2O (10 mL). The crude products were dissolved in H_2O and subjected to semipreparative RP-HPLC for purification.

Fmoc-Asp-Ala-Glu-Phe-Arg-His-Asp-Ser-Gly-Tyr(β-Ac$_4$Gal-(1-3)-*α*-Ac$_2$GalN$_3$)-Glu-Val-His-His-Gln-OH 17, 17':

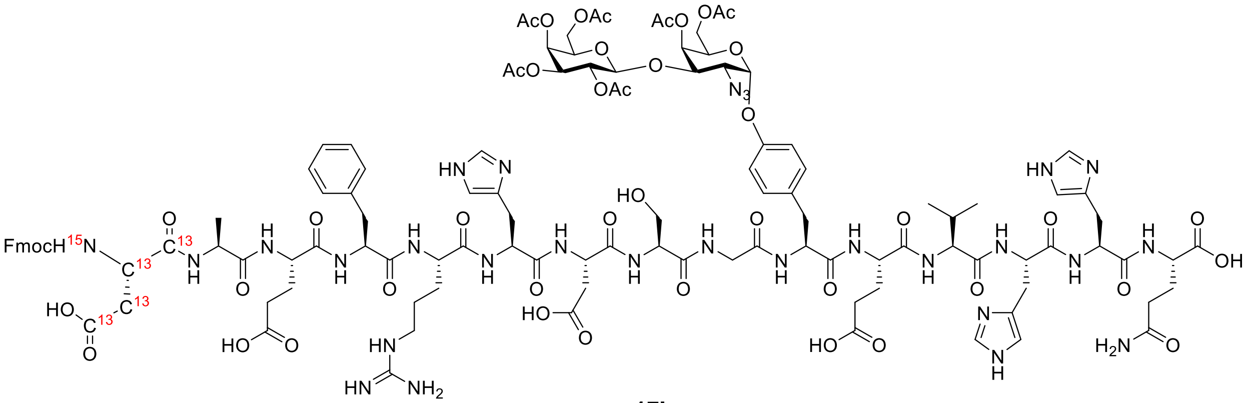

17'

Using the general method for peptide synthesis, the glycopeptide was prepared starting from the TF tyrosine building block **16** (2 eq.) and the crude was purified using SUPELCOSILTM LC-18 HPLC column (MeCN–H_2O–TFA) to get the glycopeptide **17** and **the isotopically labelled 17'** as a colorless lyophilizate in 19% and 25% yield respectively. **17: MALDI-MS**: *m*/*z* calc. for $C_{117}H_{148}N_{28}O_{44}$: 2649.02; found 2649.3. **17'**: **HRMS**: *m*/*z* calc. for $^{13}C_4C_{113}H_{148}{}^{15}NN_{27}O_{44}$: 664.7658; found 664.7711 $[M+4H]^{4+}$.

Fmoc-Asp-Ala-Glu-Phe-Arg-His-Asp-Ser-Gly-Tyr(β-Ac$_4$Gal-(1-3)-α-Ac$_2$GalNH$_2$)-Glu-Val-His-His-Gln-OH 18, 18':

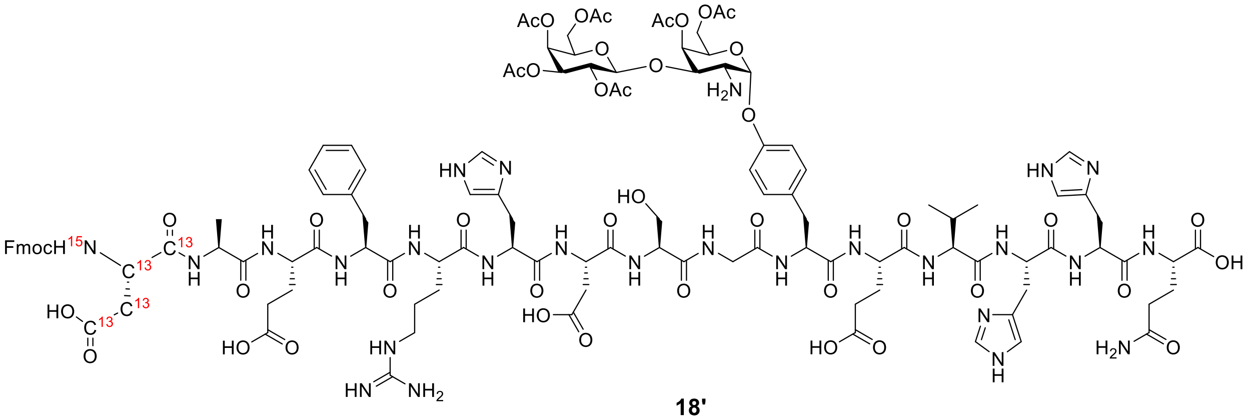

18'

The glycopeptide **17** (5 mg, 1.9 μmol) was dissolved in MeOH:AcOH:DCM (2:1:1) (1 mL) followed by addition of Zn dust (1.9 mg, 28.3 μmol). The mixture was allowed to stir for 15 min and monitored with MALDI-TOF-MS till the reactions completed. The reaction mixture was then filtered and subjected to HPLC for purification to give glycopeptide in 90% and 89% yields respectively for **18** and **18'**. **18: HRMS:** *m*/*z* calc. for $C_{117}H_{150}N_{26}O_{44}$: 657.0156; found: 657.0753; **18': HRMS:** *m*/*z* calc. for $^{13}C_4C_{113}H_{150}{}^{15}NN_{25}O_{44}$: 658.2682; found: 658.2712 $[M+4H]^{4+}$.

Fmoc-Asp-Ala-Glu-Phe-Arg-His-Asp-Ser-Gly-Tyr(β-Ac$_4$Gal-(1-3)-α-Ac$_2$GalNHAc)-Glu-Val-His-His-Gln-OH 19, 19':

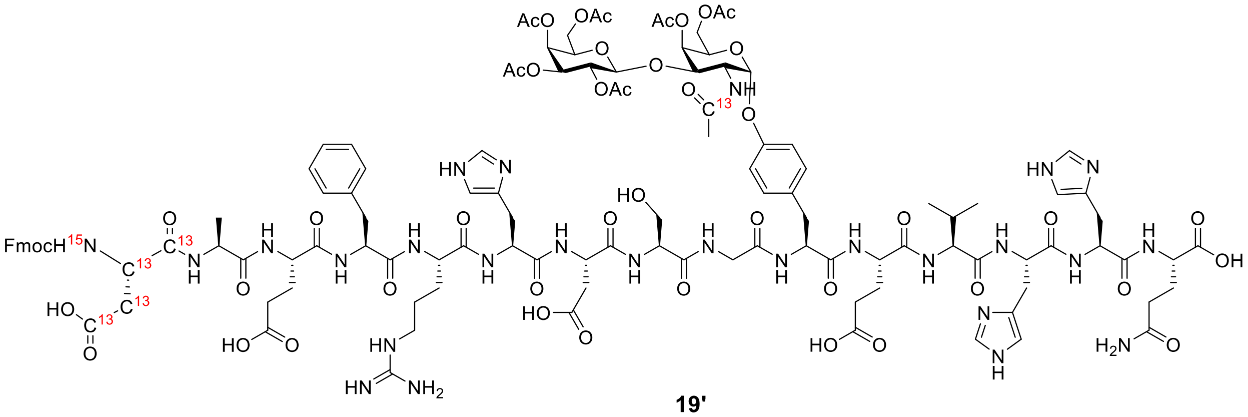

19'

Glycopeptide **18** (4 mg, 1.5 μmol) was dissolved in MeOH (300 μL), then isotopically labeled Ac_2O (50 μL) was added and stirred for 20 min. The mixture was introduced to HPLC for purification to afford glycopeptide **19** in 93% yield and **19'** in 95% yield. **19: HRMS:** 667.2674, found 667.2705 $[M+4H]^{+4}$ **: 19': HRMS:** *m*/*z* calcd for $^{13}C_4C_{115}H_{152}{}^{15}NN_{25}O_{45}$;, 668.7709, found 668.7683 $[M+4H]^{+4}$; 672.2748; found 672.3021. $[M+4H+CH_3]^{+4}$

H-Asp-Ala-Glu-Phe-Arg-His-Asp-Ser-Gly-Tyr(β-Gal-(1-3)-α-GalNHAc)-Glu-Val-His-His-Gln-OH 20, 1:

Deacylation and Fmoc deprotection of the glycopeptide **19** were taken place by dissolving the glycopeptide **19** (3 mg) in methanol and a 5% solution of NaOMe in MeOH was added slowly where pH was adjusted to around 9-10. The reaction mixture was stirred at rt for 4 h until completion as indicated by MALDI-TOF-MS. The desired product was purified using SUPELCOSILTM LC-18 HPLC column (MeCN–H_2O–TFA) to afford the glycopeptide **20** as a colorless solid in 95% yield and 92% yield for glycopeptide **1**.

20: HRMS: 548.7345; found: 548.7364 $[M+4H]^{4+}$.

1: HRMS: *m*/*z* calcd for $^{13}C_4C_{115}H_{152}{}^{15}NN_{25}O_{45}$: 550.4872, found 550.4918 $[M+4H]^{4+}$.

^{1}H-NMR ($CDCl_3$, 500 MHz) of **16**

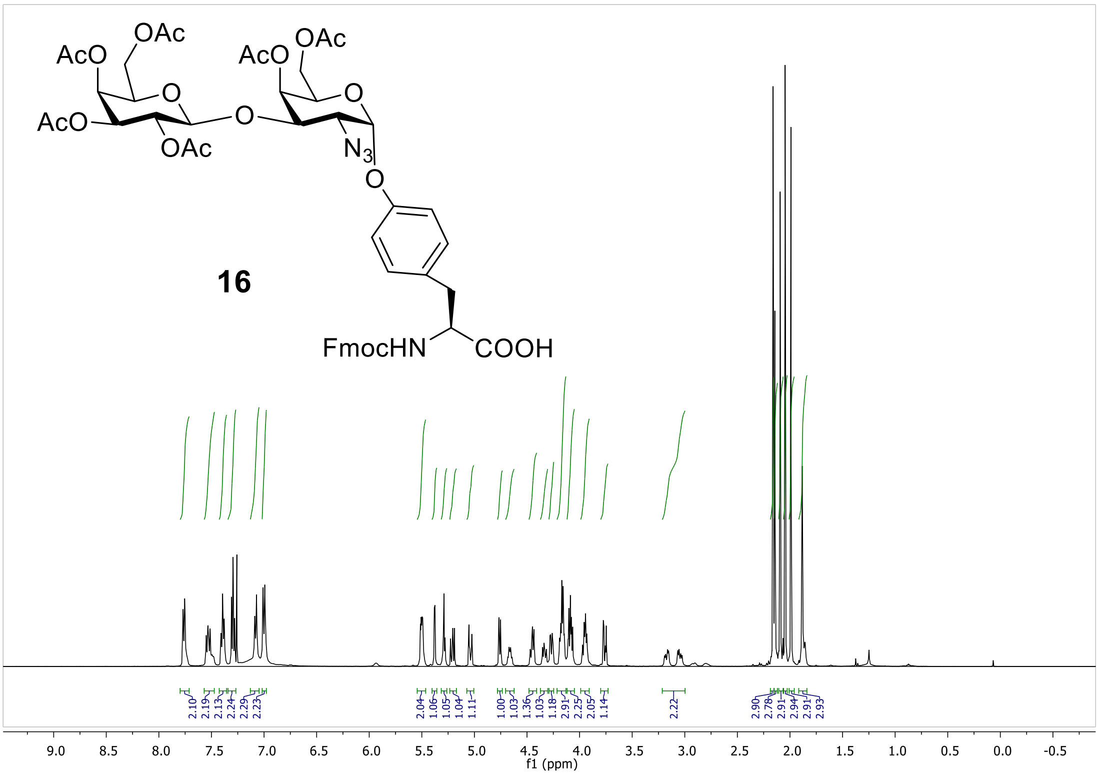

^{13}C-NMR ($CDCl_3$, 125 MHz) of **16**

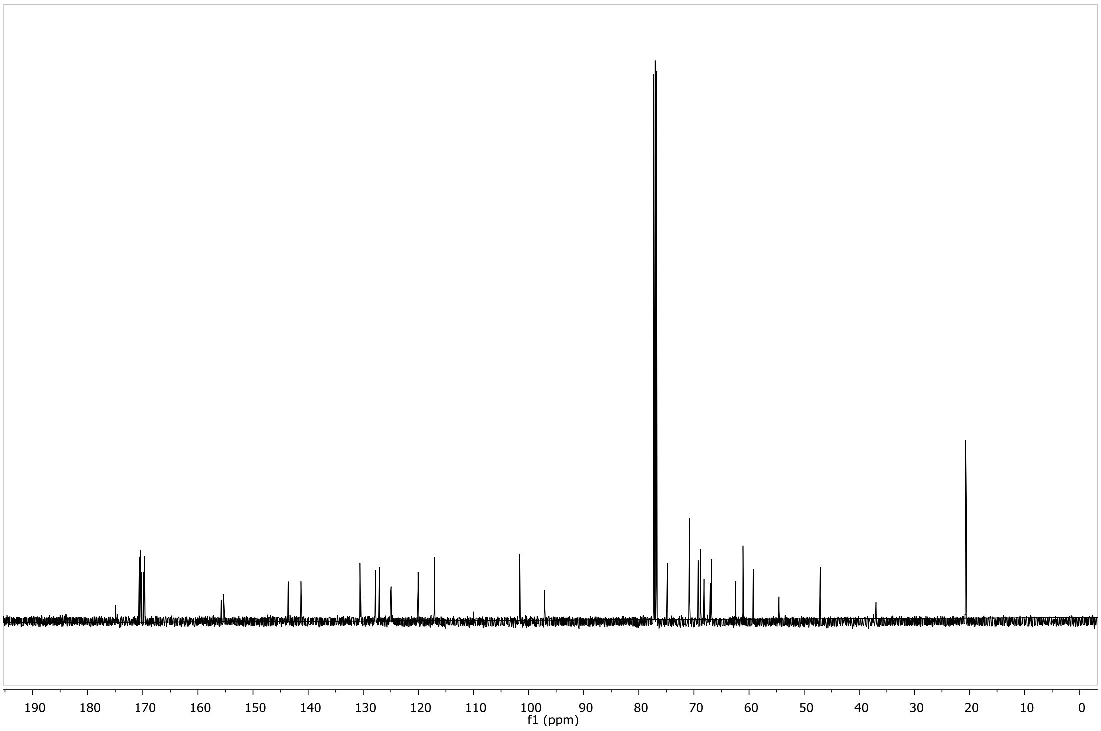

gCOSY ($CDCl_3$, 500 MHz) of **16**

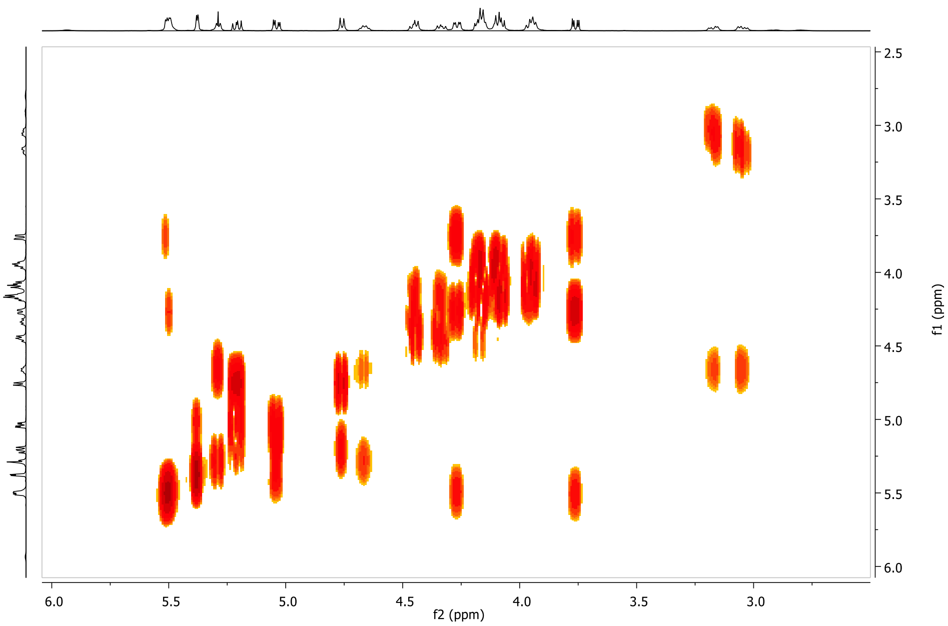

Coupled_gHSQC ($CDCl_3$, 500 MHz) of **16**

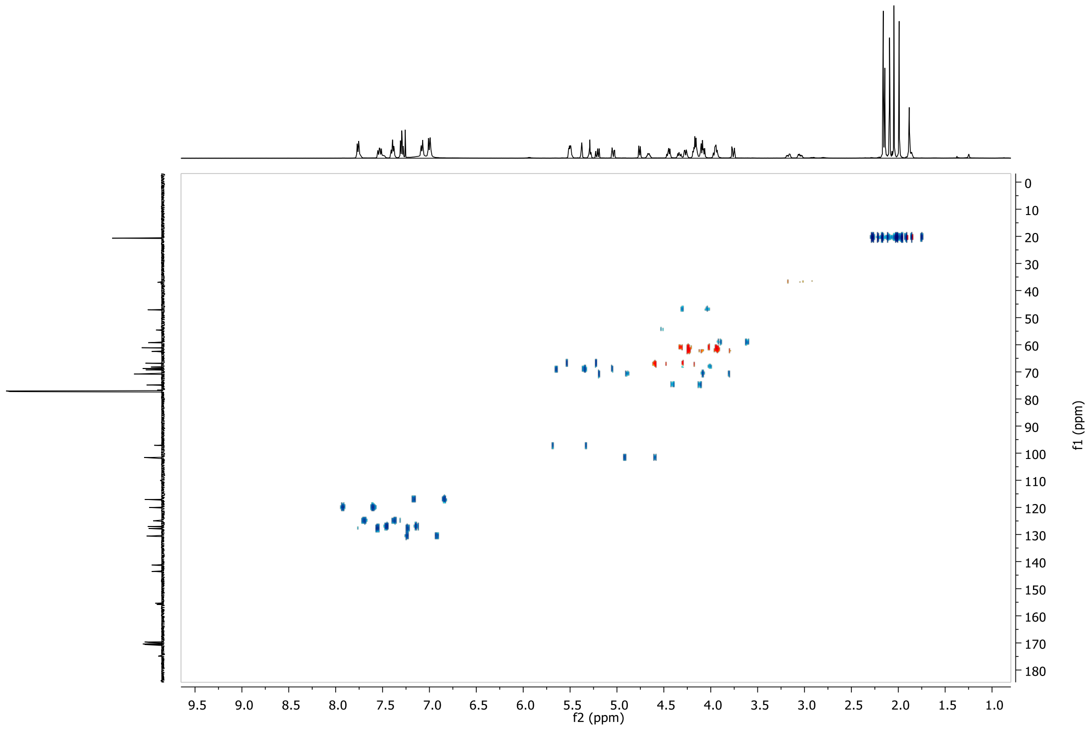

Glycopeptide **17'**:

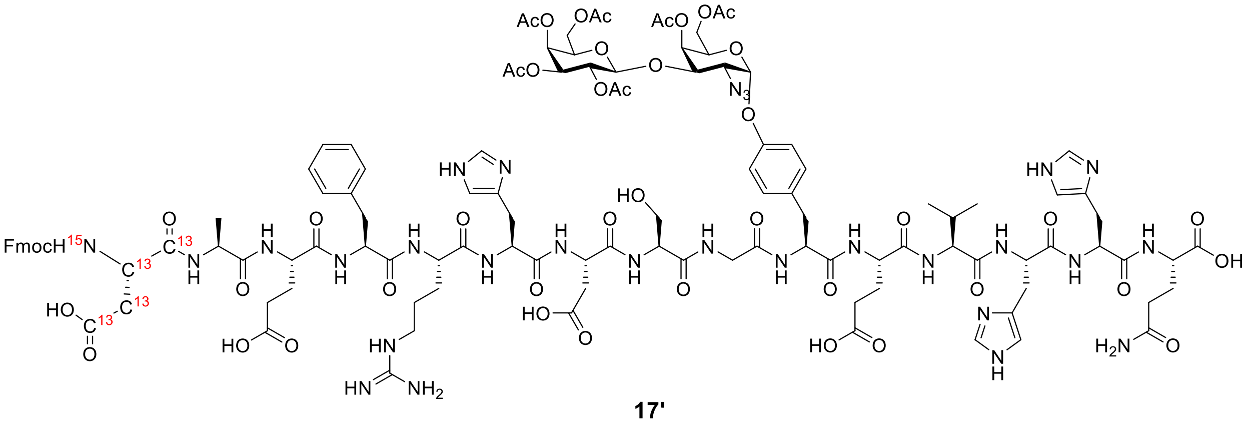

17'

HPLC chromatogram of purified glycopeptide **17'**:

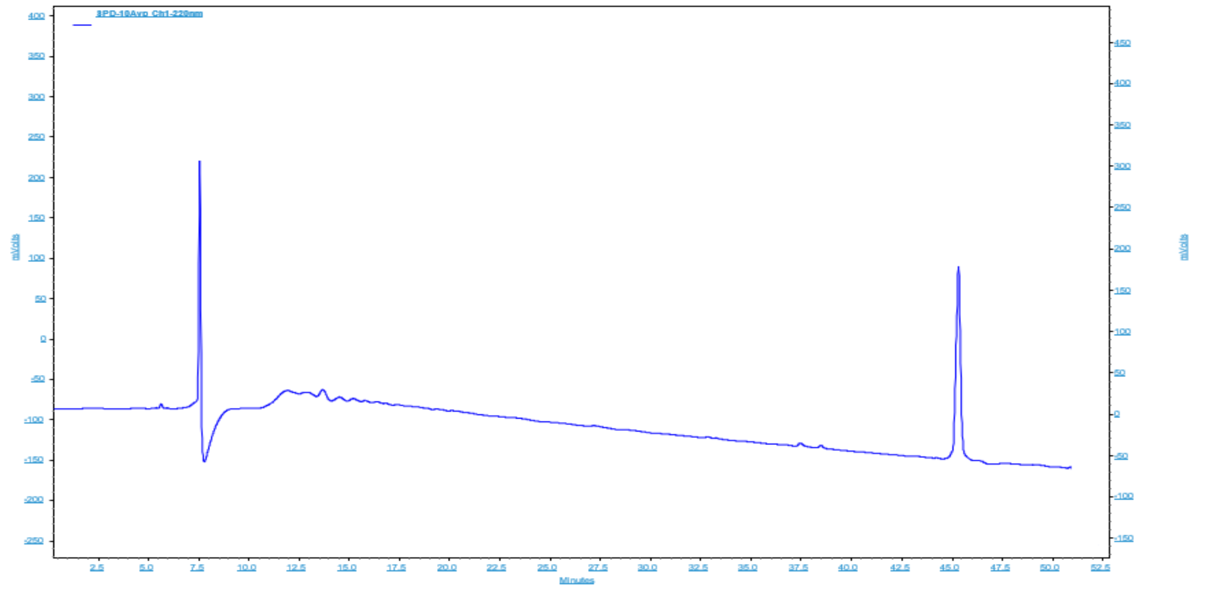

ESI-TOF-MS of glycopeptide **17'**:

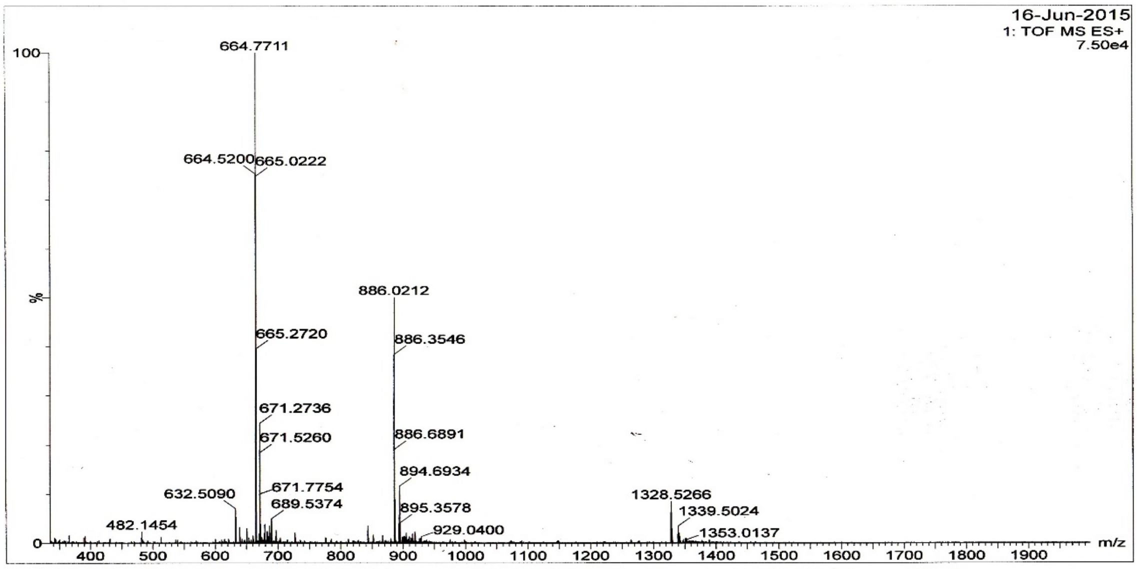

Glycopeptide **17:**

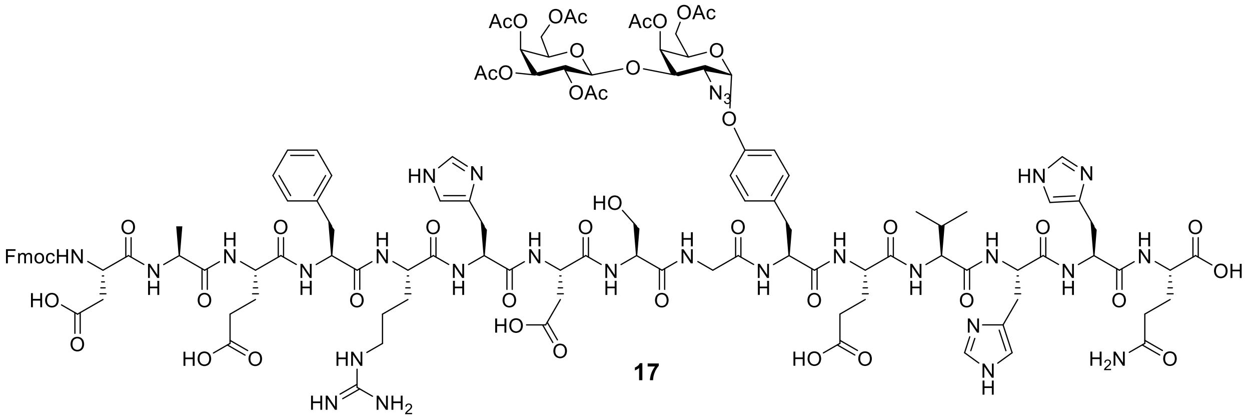

MALDI-TOF-MS of glycopeptide **17:**

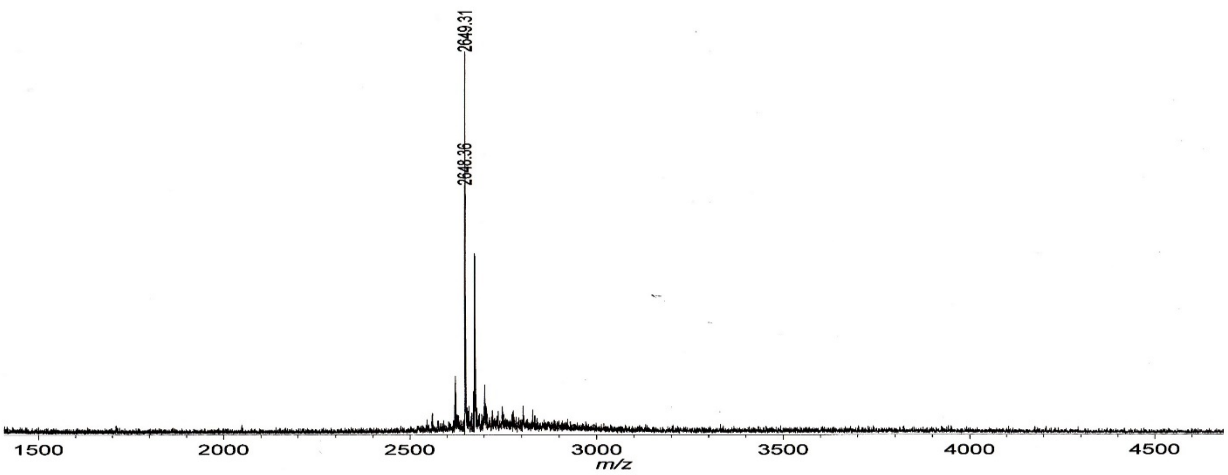

Glycopeptide **18:**

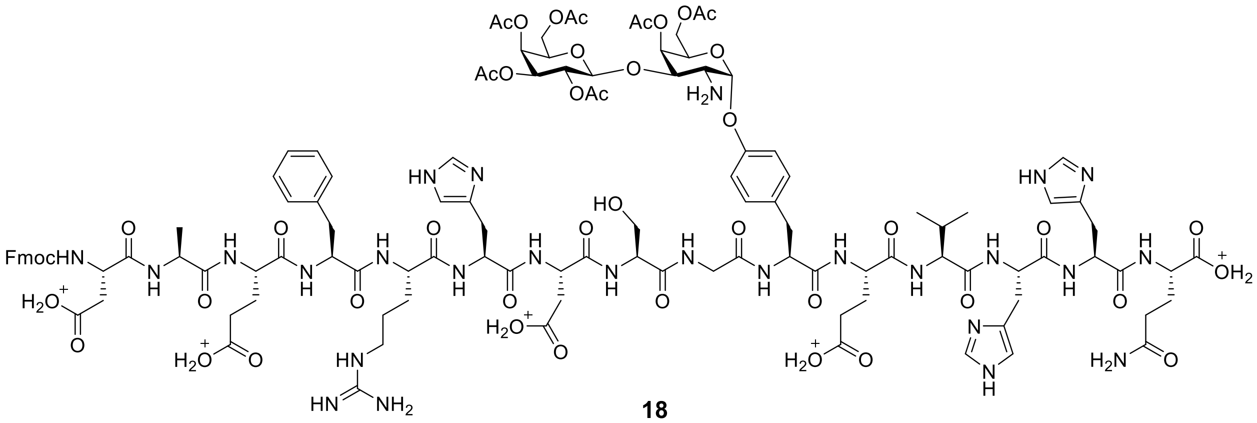

HPLC chromatogram of purified glycopeptide **18:**

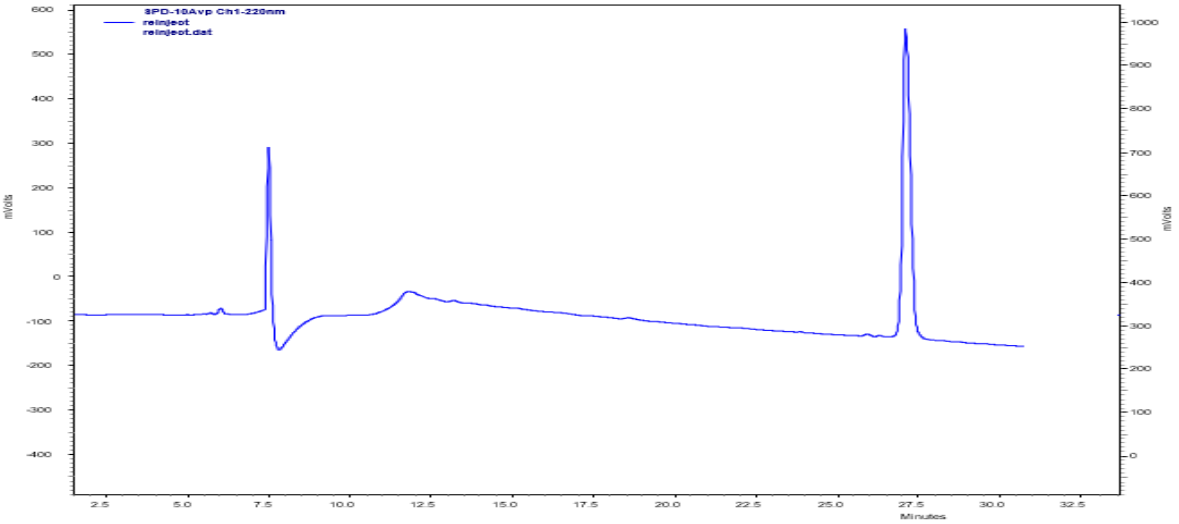

ESI-TOF-MS of glycopeptide **18:**

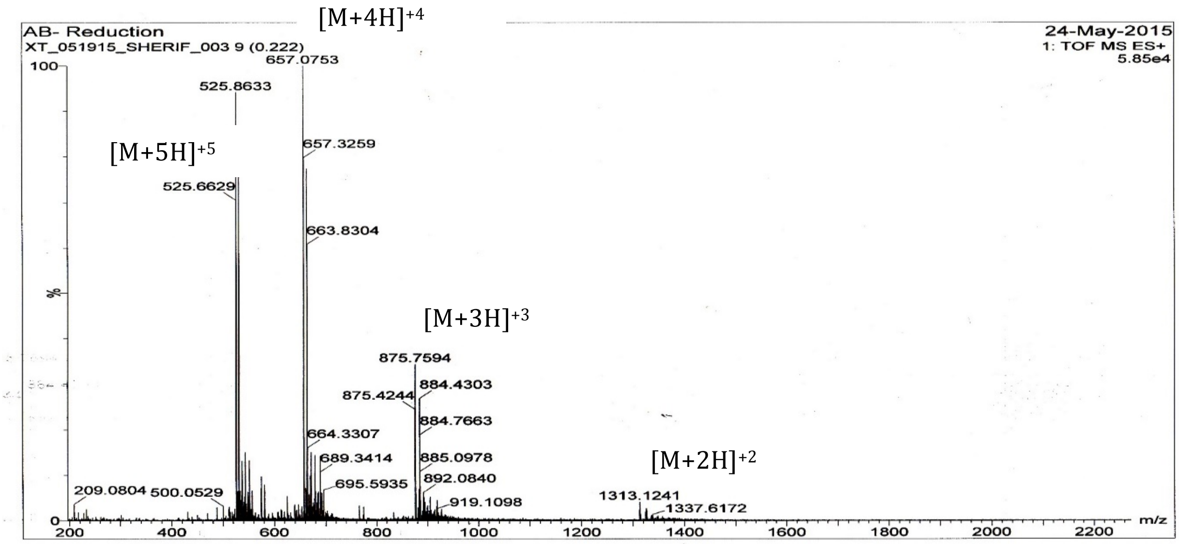

Glycopeptide **18’:**

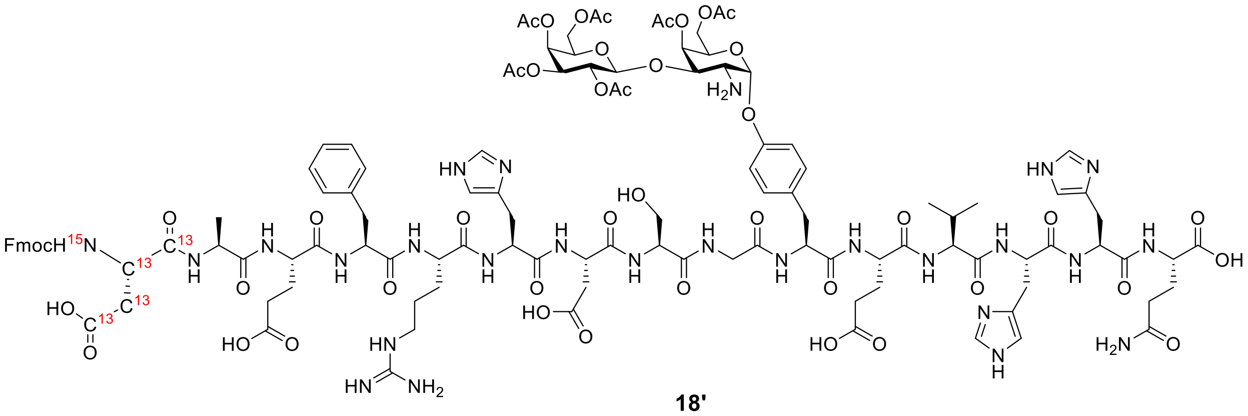

18'

ESI-TOF-MS of glycopeptide **18’:**

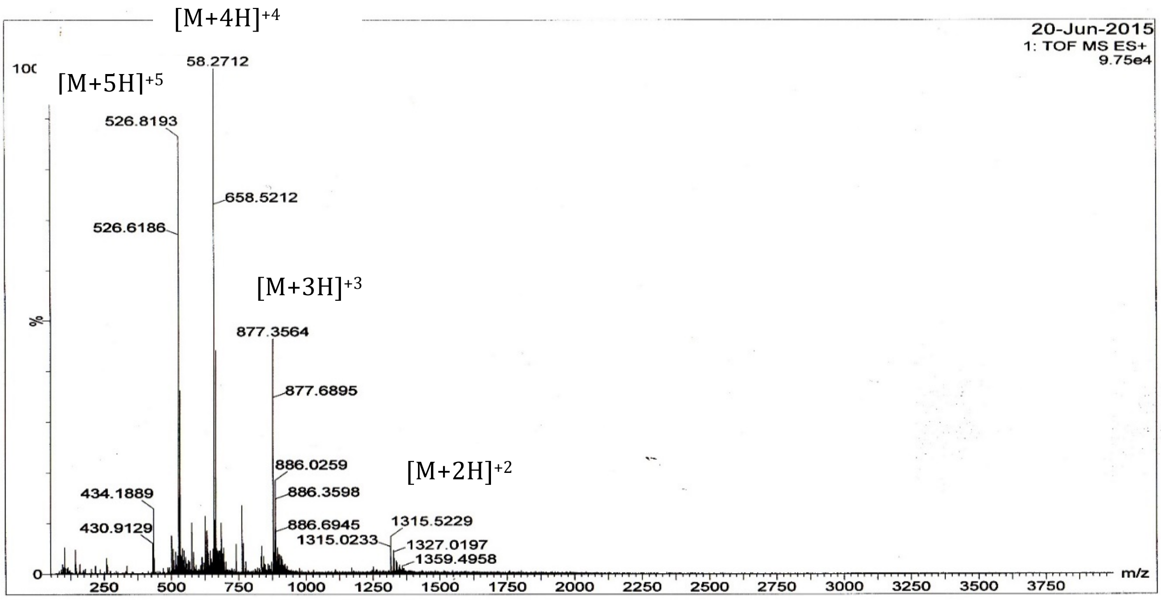

Glycopeptide **19:**

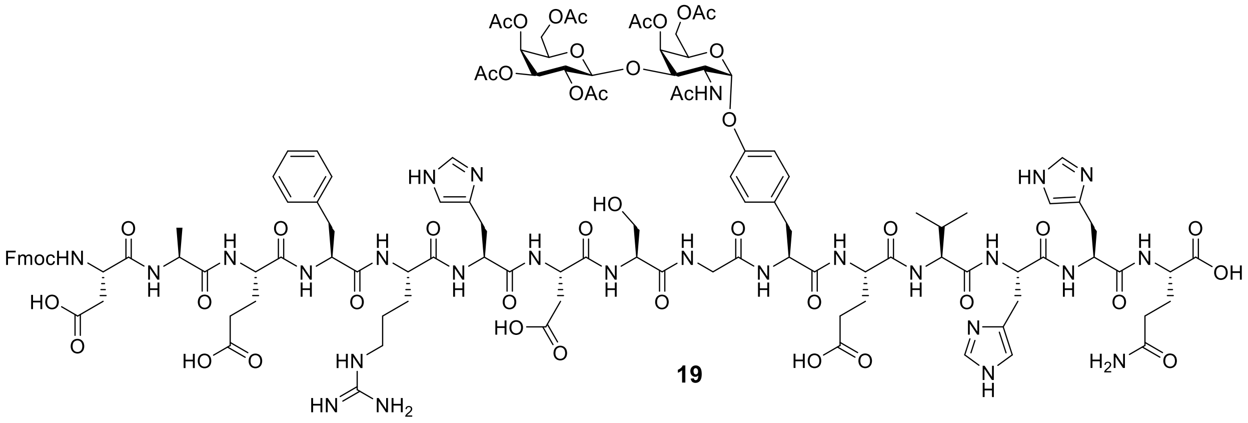

19

HPLC chromatogram of purified glycopeptide **19:**

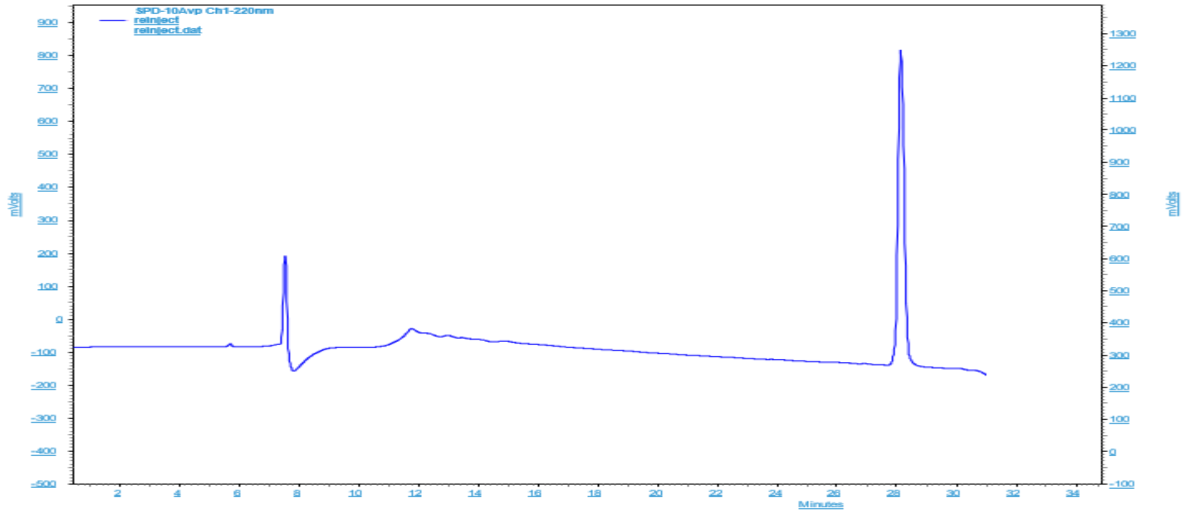

ESI-TOF-MS of glycopeptide **19:**

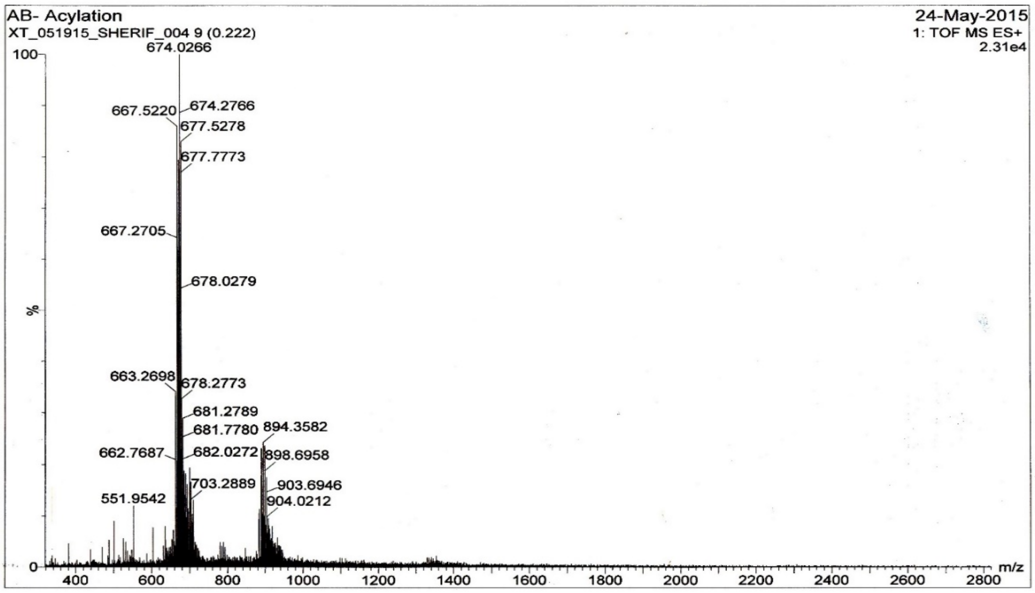

Glycopeptide **19’:**

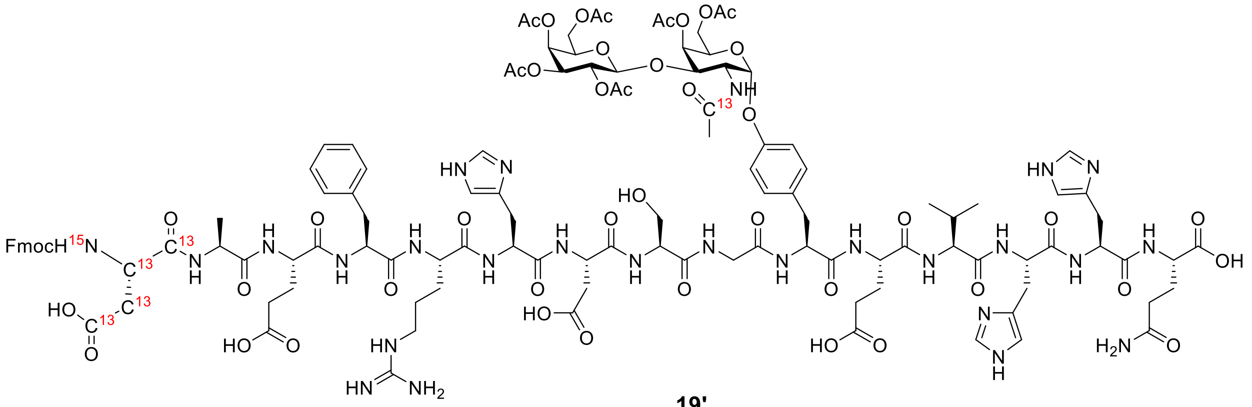

19'

ESI-TOF-MS of glycopeptide **19’:**

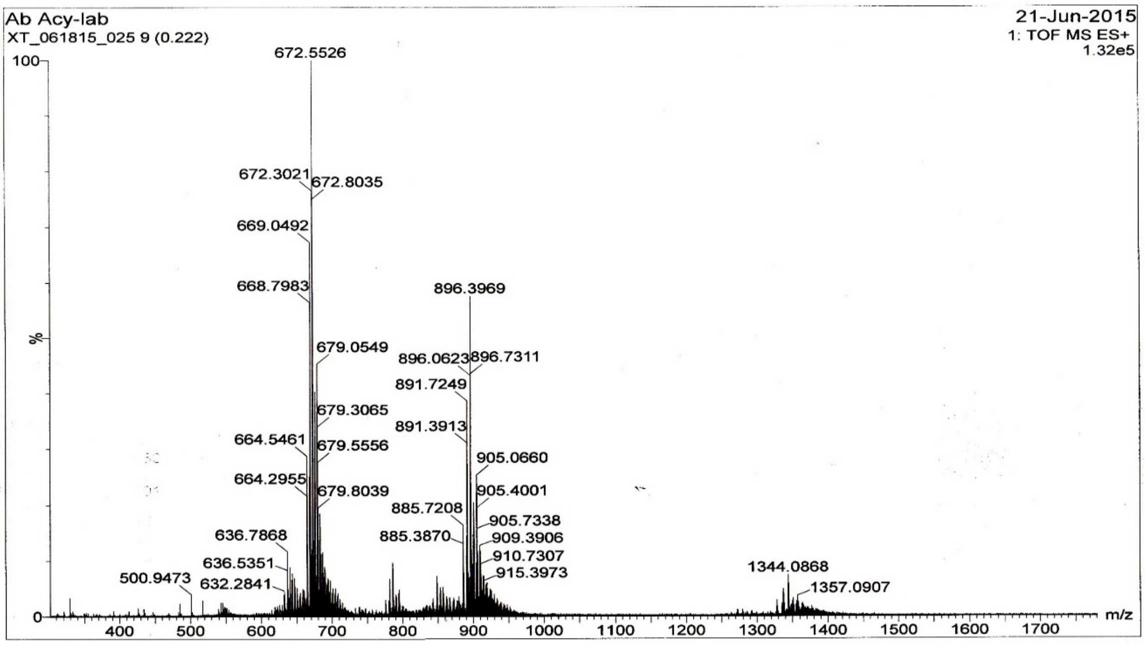

Glycopeptide 20:

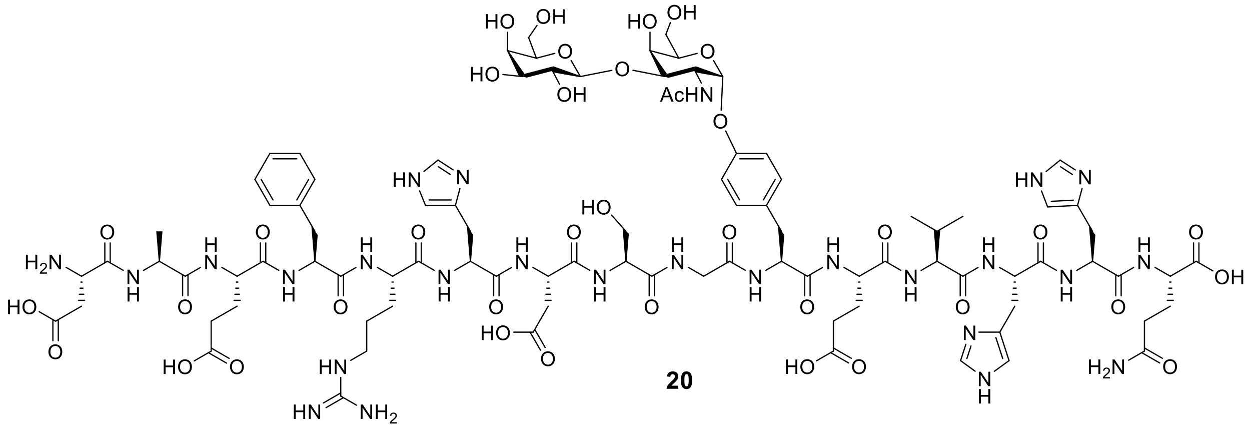

ESI-TOF-MS of glycopeptide **20:**

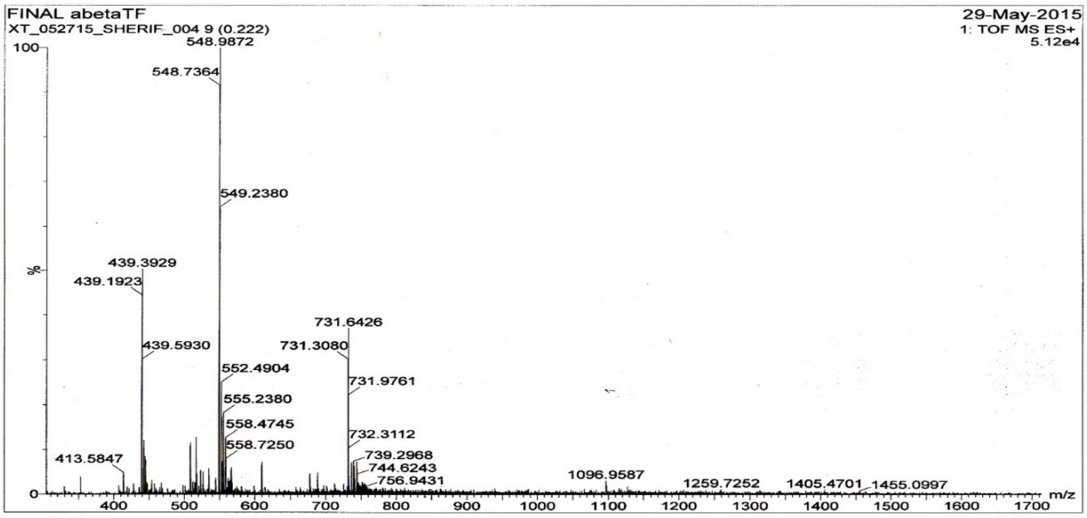

HPLC chromatogram of purified glycopeptide **20:**

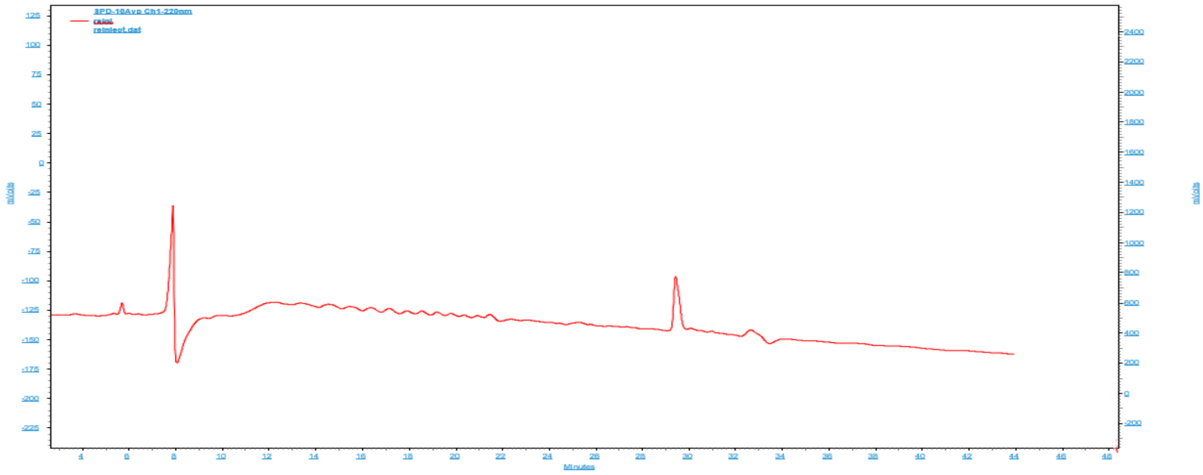

Glycopeptide 1:

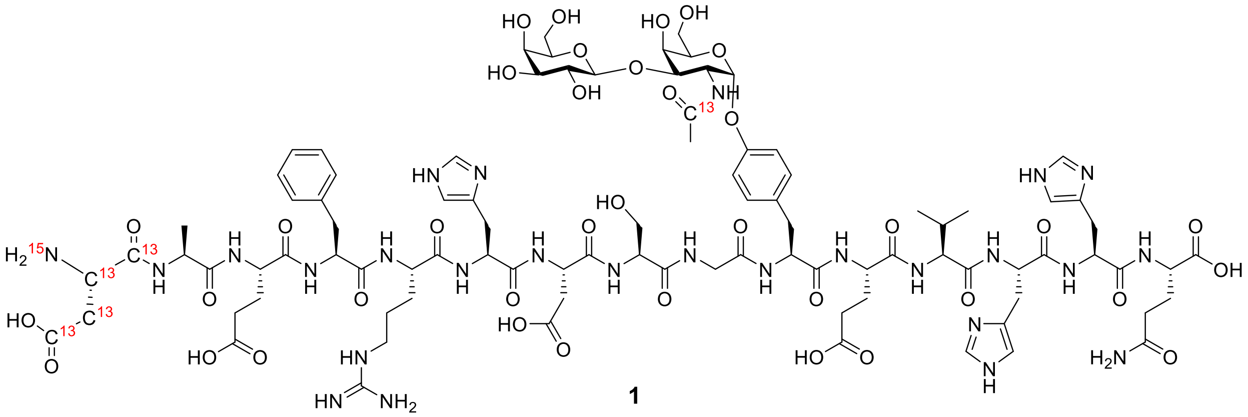

1

HPLC chromatogram of purified glycopeptide **1:**

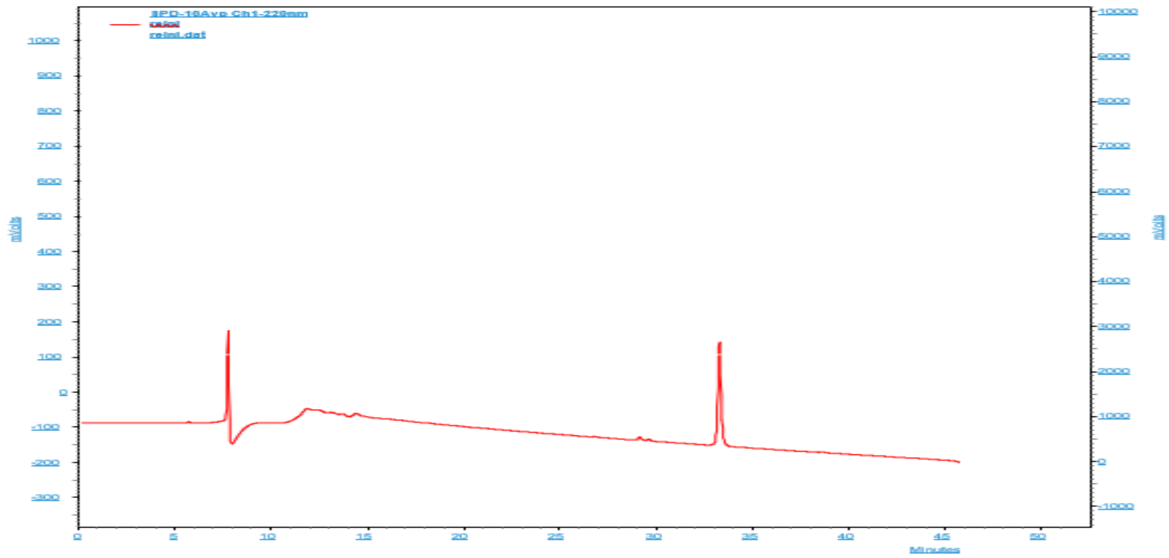

ESI-TOF-MS of glycopeptide **1:**

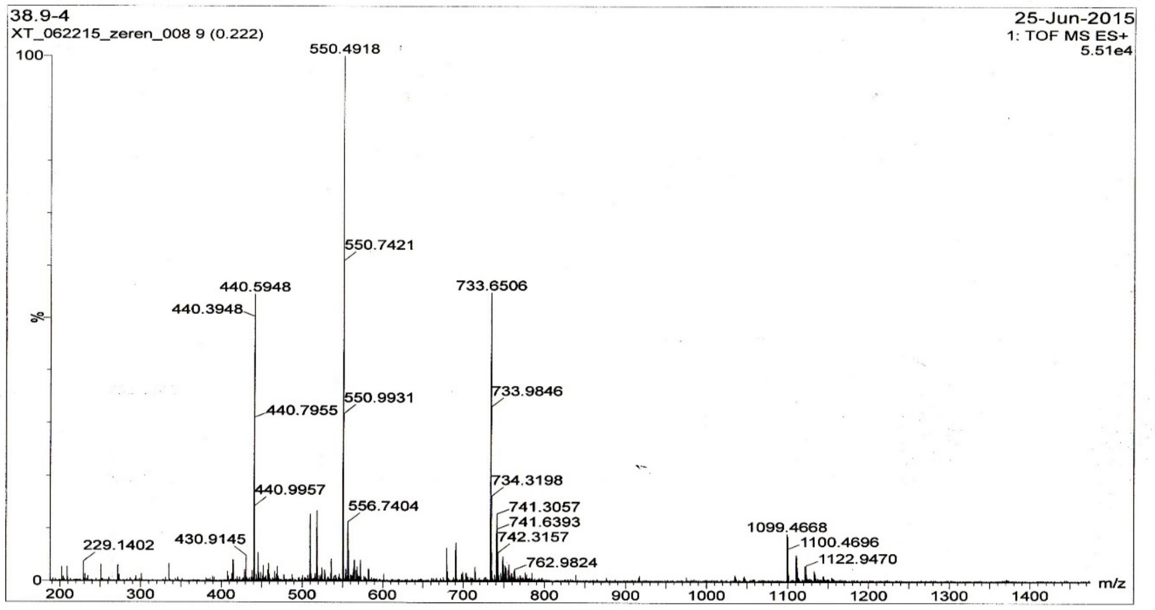

^{1}H-NMR ($CDCl_3$, 500 MHz) of **1**

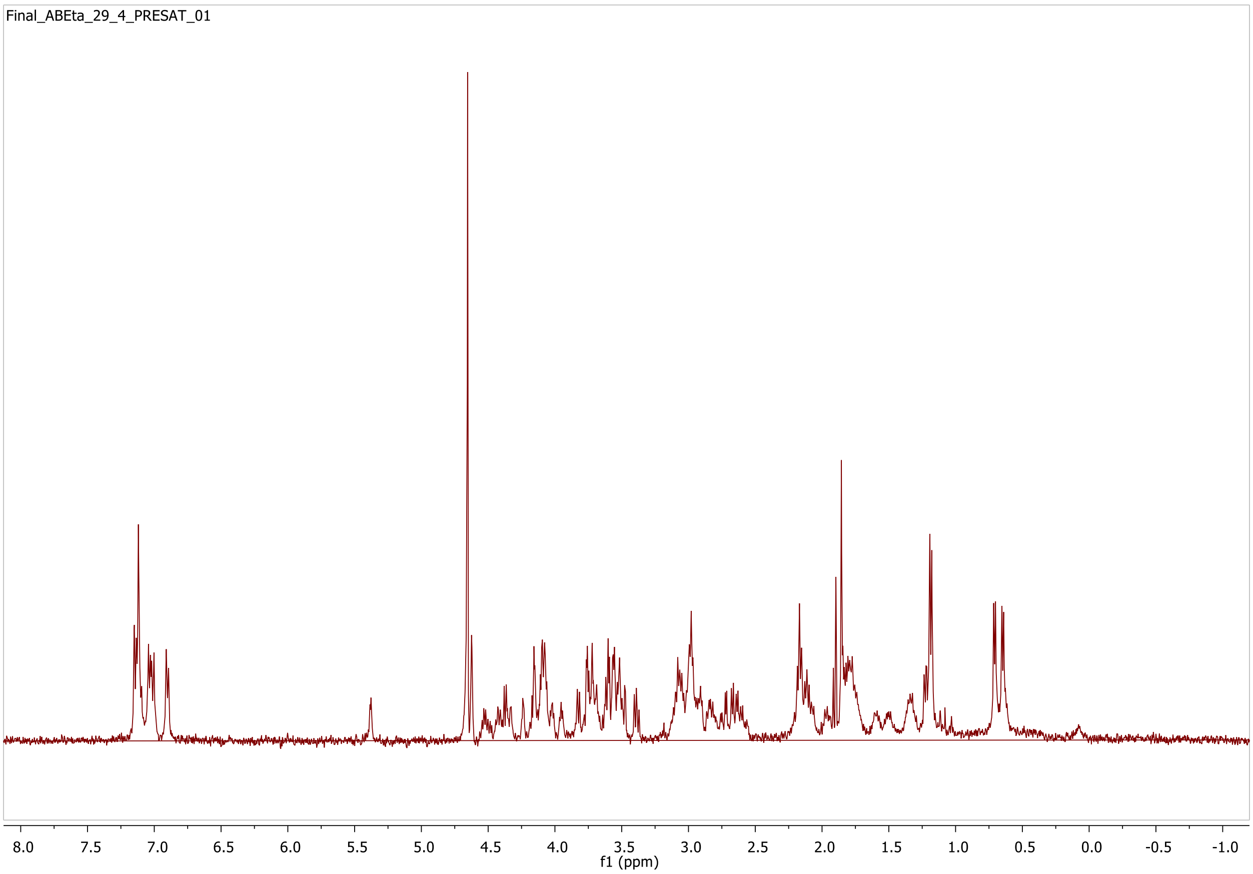

References:

1. Z. Wang, L. Zhou, K. El-Boubbou, X. S. Ye and X. Huang, *J. Org. Chem.*, 2007, **72**, 6409.

2. Pett C, et al. (2018) Effective assignment of α2,3/α2,6 sialic acid isomers in LC-MS/MS based glycoproteomics. Angew Chem Int Ed Engl. 2018, **57**, 9320-9324.
